# Synthesis of Novel Amphiphilic Fluorinated Polymers for the Dispersion of Hydrophobic Gold Nanoparticles, Quantum Dots, or Highly Fluorinated Molecules in Water

**DOI:** 10.1021/acsnanoscienceau.5c00069

**Published:** 2025-09-27

**Authors:** Galder Llorente, Juan Manuel Arango, Noelia Soto, Olena Kyzyma, Andres Alejandro Yanez Crespo, Clement Blanchet, Aitzol Garcia-Etxarri, Marité Cárdenas, Monica Carril

**Affiliations:** † 226245 Donostia International Physics Center, Paseo Manuel de Lardizabal 4, Donostia-San Sebastian 20018, Spain; ‡ Instituto Biofisika (UPV/EHU, CSIC), University of the Basque Country, Barrio Sarriena s/n, Leioa 48940, Spain; § Departamento de Bioquímica y Biología Molecular, University of the Basque Country (UPV/EHU), Barrio Sarriena s/n, Leioa 48940, Spain; ∥ 203232 Fundación Biofísica Bizkaia/Biofisika Bizkaia Fundazioa (FBB), Barrio Sarriena s/n, Leioa 48940, Spain; ⊥ 128803 European Molecular Biology Laboratory EMBL, Hamburg Unit, c/o DESY Notkestrasse 85, Hamburg 22603, Germany; # IKERBASQUE, Basque Foundation for Science, Plaza Euskadi 5, Bilbao 48009, Spain

**Keywords:** fluorinated polymers, NP polymer coating, QDs fractals, ^19^F MRI contrast agents, SAXS

## Abstract

The transfer of inorganic nanoparticles (NPs) into water is usually considered a challenge, as NPs are preferably synthesized in organic solvents and commonly bear hydrophobic ligands. Consequently, various methods have been reported to achieve their transfer to aqueous media. Among these, a polymer coating using amphiphilic polymers represents a particularly useful approach. These polymers can interact with the NP surface via their hydrophobic moieties, while their hydrophilic side remains exposed to the aqueous media, thus enabling dispersion in water. In this paper, we present the facile synthesis of several fluorinated, hydrosoluble amphiphilic polymers, and we study the coating of different types of metallic NPs, such as gold nanoparticles and quantum dots (QDs). Gold NPs were transferred via a phase transfer protocol, but for more sensitive QDs, we used the film hydration method. For QDs, the high hydrophobicity of fluorinated moieties on the polymer was particularly advantageous in repelling water and preserving the optical properties of QDs. Fractal arrangements in aqueous solution for polymer-coated QDs were analyzed by small-angle X-ray scattering (SAXS) but also observed by TEM. Additionally, we employed these fluorinated polymers to transfer two highly hydrophobic and fluorinated molecules (PERFECTA and PFCE), commonly used as contrast agents in ^19^F magnetic resonance imaging (^19^F MRI), into aqueous media. We evaluated their transverse and longitudinal relaxation times to assess their suitability for use as contrast agents for ^19^F MRI.

## Graphical abstract

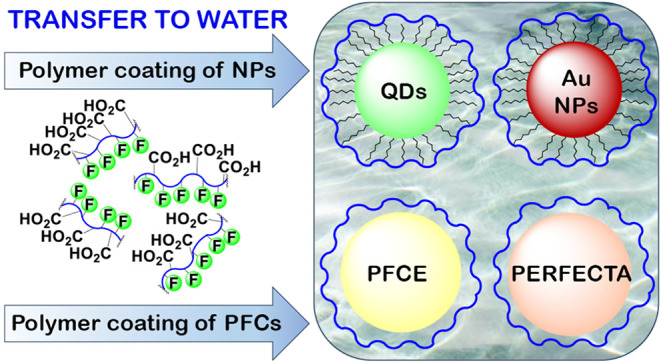

## 1 Introduction

Inorganic or metallic nanoparticles (NPs) are frequently synthesized in organic solvents, and the so-obtained NPs are most of the time of hydrophobic character.^1^ For certain applications, such as those in the field of nanomedicine,^2,3^ it is essential to ensure that these NPs remain colloidally stable in aqueous solutions, and there are currently several strategies to do so. One possible approach is the modification of the ligands directly attached to the NP surface by using hydrophilic ligands such as poly­(ethylene glycol) (PEG)-based groups. PEGylated ligands are multifaceted. Due to their good solubility in both organic and aqueous solvents, they facilitate the NP transfer to the water phase.^4^ One possibility is the substitution of the NP surface ligands by another type of hydrophilic ones, using the principle of ligand exchange in the presence of an excess of a water-soluble ligand.^5^ Another approach is based on using an amphiphilic polymer to coat the NP surface. In particular, such amphiphilic polymer contains a hydrophobic side that wraps around the hydrophobic NP, while its hydrophilic side remains exposed to the surrounding medium, thereby rendering the entire NP water-soluble.^6^ Very frequently, the hydrophobic side of the polymer is functionalized with long alkyl chains of 12 or more carbon atoms, looking for intercalation of those alkyl chains in between the NP ligands to facilitate and stabilize the resulting polymer coating.

The efficiency of the transfer-to-water protocols for some NP types that may be sensitive to water or surface oxidation is critical to prevent nanomaterial degradation and loss of their physical properties. This is key for NP types such as quantum dots (QDs) or upconverting NPs, which may have their optical properties affected upon transfer to water.^7^ Quantum dots exhibit significant advantages for cellular labeling and in vivo imaging compared to standard organic dyes owing to their intense photoluminescence (PL) and resistance to photobleaching.^8,9^ However, wider applications of QDs to intracellular imaging and sensing have been hampered in part by the loss of their optical properties once they are transferred to aqueous solutions. In these particular cases, the contact with water results in a loss of fluorescence intensity, so the use of an amphiphilic polymer with a very hydrophobic side might be of interest to keep water away from the most sensitive NP cores. The highest hydrophobicity is achieved by the inclusion of fluorinated monomers on a polymer chain.^10^

In this contribution, we present novel amphiphilic polymers with fluorinated short chains on the hydrophobic side for a simple transfer-to-water protocol leading to NPs solubilization. The presence of fluorine, as it is so hydrophobic, results in polymers that are particularly efficient in repelling water from the surface of the wrapped NPs. Fluorinated polymers are well-established in scientific literature^11^ for their use in durable surface coatings because their highly fluorinated surfaces provide excellent antifouling properties.^12^ In addition, the presence of fluorine alters the properties of the coated surfaces such as their wettability, friction, or adhesion. Fluorinated polymers have also been used as contrast agents in ^19^F magnetic resonance imaging (^19^F MRI) in the field of biomedicine.^13^ However, their use has been challenging until now since, on the one hand, fluorinated probes for ^19^F MRI require a high local concentration of fluorine atoms, which can be easily achieved with a polymer, while, on the other hand, considerable synthetic efforts are typically needed to overcome the hydrophobicity of fluorine to create water-soluble fluorinated polymers. Additionally, the spatial arrangement of these fluorinated polymers in aqueous environments often results in short transverse relaxation times that are not always ideal for ^19^F MRI applications.^14^

The polymer synthesis we propose is extremely simple, based on a known modification of a commercially available poly anhydride polymer,^15^ with a small fluorinated building block prepared by us.^16^ The protocol was adapted to include molecules other than those containing fluorinated groups to further modify the surface and the physicochemical properties of the polymer. We initially tested the polymer coating strategy with gold NPs due to their ease of preparation and handling. Their distinct red color also facilitated the visual monitoring of the transfer-to-water protocol. We further tested the polymer coating using our fluorinated polymers with more challenging QDs and verified that the quantum yield remained mostly unchanged for at least 5 days in aqueous solution. To the best of our knowledge, this is the first time that fluorinated polymers have been used for the polymer coating of NPs to transfer them to the aqueous phase and keep them colloidally stable. Additionally, we used our fluorinated polymers to bring highly fluorinated molecules, such as perfluorocarbons,^17,18^ into the water phase and measured their transverse and longitudinal relaxation times to evaluate their potential as contrast agents in ^19^F MRI.

## 2 Materials and Methods

### 2.1 General Remarks

All of the reagents were purchased and used as received. Poly­(isobutylene-*alt*-maleic anhydride) (PMA) was estimated to contain 39 repeating monomers, based on the molecular weight provided by the commercial supplier. Trioctylphosphine oxide (TOPO) capped CdSe/ZnS QDs (*d*_c_ = 6.2 ± 1.0 nm) for polymer coating were purchased from Plasmachem (Cat. Nr. PL-QD-O-530). Dodecanethiol-capped Au NPs (*d*_c_ = 4.3 ± 0.8 nm) were prepared according to literature protocols.^1^ All air- or moisture-sensitive reactions were performed under a nitrogen atmosphere. ^1^H NMR, ^13^C NMR, and ^19^F NMR were recorded on a Bruker AC-300 instrument (300 MHz for ^1^H, 75.4 MHz for ^13^C, and 283 MHz for ^19^F) at 20 °C. 2D NMR spectra were acquired on a Bruker AC 500 instrument (500 MHz for ^1^H and 125.7 MHz for ^13^C). Dynamic light scattering (DLS) measurements were recorded on nanoZS Zetasizer from Malvern at 173°. Inorganic core radii of gold NPs and QDs were obtained from the analysis of TEM images. TEM experiments were performed in a JEOL 1400 Plus transmission electron microscope operated at 100 kV, and digital images were acquired with a Hamamatsu Flash sCMOS digital camera. A sample droplet was deposited on top of a carbon-coated grid previously hydrophilized. For negative staining experiments, 2 μL of each sample was adhered for 1 min on carbon-coated grids (CF300-Cu, EMS) previously hydrophilized by glow discharge (Leica ACE200, 30 s at 5 mA). After removing the excess liquid by blotting with filter paper, grids were negatively stained using 1% uranyl acetate for 45 s. Size measurement was performed with the free software ImageJ, and size distribution analysis and histogram production were done with Origin software. Photoluminescence spectra were acquired using a Horiba Fluoromax-4 spectrophotometer, exciting at 350 nm, with 1.5 nm slits and 1 nm increments. Photoluminescence quantum yield measurements (PLQY) were recorded with an integrating sphere measurement system from Horiba, using the FluoroMax-4 spectrophotometer. The data were analyzed using PLQY measurement software FluorEssence.

### 2.2 Synthesis of **PMA-XF**

*Typical procedure*. Poly­(isobutylene-*alt*-maleic anhydride) **PMA** (11.1 mg, 0.07 mmol) in anhydrous THF (2 mL) was mixed with 1.1–0.5 equiv of fluorinated building block **F** (50–118 mM in CH_2_Cl_2_), depending on the degree of fluorination desired, in a glass vial with a magnetic stirrer (see Table S1 in the Supporting Information). The mixture was capped, and it was allowed to stir overnight at room temperature. The next morning, the vial was uncapped, and the solvent and unreacted **F** were allowed to evaporate under the hood or with the help of compressed air to afford a white powder. The so-obtained polymers were further dried under vacuum before characterization. A small portion of solid polymer was dissolved in NaOH (1 M) and analyzed by ^1^H NMR, ^19^F NMR, and HSQC (see section 2.2 in the Supporting Information). The solution concentration and the degree of fluorination were calculated from ^19^F NMR signal integration (broad bands between −72 and −71 ppm) using a known amount of trifluoroacetic acid (TFA) as a reference compound.

### 2.3 Synthesis of **PMA-XF/SO**_**3**_

*Typical procedure*. A solution of *tert*-butyl (2-aminoethyl)­carbamate (0.5 M in THF, 0.6–0.1 equiv) was added to a solution of **PMA** (11.1 mg, 0.07 mmol) in anhydrous THF (4 mL). After vigorous stirring for 2 h at r.t., a solution of **F** (118 mM in DCM, 1–0.4 equiv) was added (see Table S1 in the Supporting Information). The solution was vigorously stirred overnight. The next morning, the solvent was evaporated first with compressed air and then under a vacuum. Subsequently, the so-obtained solid was dissolved in DCM (1 mL), followed by the careful addition of HCl (4 M in dioxane, 57 equiv to the theoretical Boc content). After vigorous stirring for 6 h at r.t., the solvent was evaporated with compressed air followed by vacuum. The disappearance of the Boc group and the obtention of the free primary amine were confirmed by ^1^H NMR. The resultant powder was dissolved in a Na_2_CO_3_ solution (1 M, 2.1 mL), followed by the addition of sodium 3-bromopropane-1-sulfonate (5 equiv to the theoretical amount of primary amine). The solution was vigorously stirred for a further 24 h. The crude mixture was purified using a desalting column (PD-10 Sephadex G-25 M). After lyophilization, **PMA-XF/SO**_**3**_ **Na** with different degrees of fluorination was isolated as a white powder. The polymers were identified by ^1^H NMR, ^19^F NMR, and HSQC (see section 2.2 in the Supporting Information).

### 2.4 Polymer Coating of Dodecanethiol-Capped Gold NPs (*d*_c_ ≈ 4 nm)

*Typical procedure:* A clear solution of fluorinated polymer **PMA-XF** (*R* = 50 or 75, 3 or 5 mM, respectively) was prepared by dissolving the polymer in aqueous NaOH (approximately 0.1 M) and sonicating the solution in a bath for 10 min at 37 kHz and 30 °C. The latter polymer solution was added onto a solution of gold NPs (0.6 μM in DCM, 1 mL) placed in a vial with a magnetic stirrer and a screw cap. The two insoluble layers were vigorously stirred, and after about 20–30 min of stirring, a transfer of the red color from the organic layer to the aqueous layer was already observed, which evidenced the phase transfer. The mixture was allowed to stir overnight to allow proper capping and homogenization of the sample. The next morning, the reaction mixture was transferred to an Eppendorf container and shortly centrifuged to improve phase separation. The red colored aqueous phase with **AuNPs@PMA-XF** was collected, and the organic phase was further washed twice with water to recover all water-soluble NPs. Aqueous extracts were joined together and purified by centrifugation (9 × 10^4^ g, 25 °C, 30 min, 3 times), washing with water to remove excess **PMA-XF**. The pellets containing **AuNPs@PMA-XF** were reconstituted in 2 mL of water and filtered (0.22 μm) to be used for characterization purposes by UV–vis, TEM, and Zeta potential.

### 2.5 Polymer Coating of TOPO-Capped CdSe/ZnS QDs (*d*_c_ ≈ 6 nm)

*Typical procedure:* A solution of CdSe/ZnS QDs (0.95 μM) in chloroform (1 mL) was placed in a vial. Then, the fluorinated polymer **PMA-100F** (*R* = 28, 2.2 mg) was added as a solid, and the so-obtained solution was sonicated in a bath for 10 min at 37 kHz and 20 °C. Subsequently, the solution was vigorously stirred for 30 min at r.t., and the chloroform was smoothly evaporated under the hood using compressed air. Then, NaOH (1 mL, 0.1 M) was rapidly added, the mixture was sonicated in a bath for 5 min at 37 kHz and 20 °C, and vigorously stirred for an additional 15 min at r.t. The water-dispersed QDs were purified by centrifugation (9 × 10^4^ g, 20 °C, 30 min, 2 times) by washing with water to remove polymer residues without QDs. The pellets containing **QDs@PMA-100F** were reconstituted in 0.5 mL of water to be characterized by TEM and fluorimetry.

### 2.6 Encapsulation of PFCE

*Typical procedure*. A clear solution of fluorinated polymer **PMA-100F** (8.9 mg, 0.02 M, final concentration) was prepared by sequentially adding aqueous NaOH (200 μL, 0.1 M), followed by NaOH (20 μL, 5 M) and water (540 μL), and sonicating the mixture in a bath for 10 min at 37 kHz and 30 °C. 190 μL of the latter polymer solution was added onto different Eppendorfs containing different amounts of PFCE (from 153.5 to 15.35 μmol). The so-obtained heterogeneous solutions were sonicated in a bath for 5 min at r.t., vortexed for 5–10 s, and further sonicated for 20 min at 40 °C. Then, the solutions were alternately briefly hand-shaken and sonicated for a further 10 min at 40 °C to achieve an emulsion that separated over time. After 2 h standing at r.t., 2 phases and a turbid interphase were formed. For ^19^F NMR analysis, the upper clear phase was taken, and the concentration of PFCE was measured using a coaxial insert loaded with TFA (0.024% v/v) in deuterium oxide. Subsequently, the upper layer was filtered through a 0.45 μm filter and analyzed again by ^19^F NMR analysis. Filtered and nonfiltered samples were characterized by DLS, and the transverse and longitudinal relaxation times for all of the peaks of interest were measured (see section 4 in the Supporting Information).

### 2.7 Encapsulation of PERFECTA

*Typical procedure*. **PMA-100F** (5.0 mg, 0.01 mmol), PERFECTA^19^ (10 mg, 0.009 mmol), and chloroform (1.5 mL) were added to a vial and stirred overnight at 80 °C. After this time, the chloroform was slowly evaporated under the hood, assisted by a stream of N_2_. Afterwards, NaOH (1.0 mL, 0.1 M) was added to the resulting solid, and it was further stirred at 80 °C for 2 h, obtaining a slightly cloudy suspension. To change the medium from NaOH (0.1 M) to water or sodium bicarbonate, 10 kDa MWCO centrifugal filters were used, and samples were centrifuged in cycles of 5 min at 1.4 × 10^4^ g, until the expected pH was obtained. The concentration of PERFECTA was measured by ^19^F NMR using a coaxial insert loaded with TFA (0.077% v/v) in deuterium oxide (see section 5 in the Supporting Information).

### 2.8 Small-Angle X-ray Scattering (SAXS) Experiments

SAXS measurements were performed at the P12 beamline of EMBL, PETRA III (DESY, Hamburg).^20^ Automated sample changer at 25 °C was used to load 40 μL of sample into an in-vacuum, flow-through capillary. An X-ray beam with λ = 0.124 nm (photon energy E = 10 keV) was employed to measure aqueous dispersions of QDs at 2 concentrations (1 and 0.5 μM). Scattered photons were collected on a photon-counting PILATUS 6 M detector with a sample-to-detector distance of 3 m, resulting in a scattering vector range of 0.02 and 7.25 nm^–1^ (*q* = 4­(π/λ) (sin θ/2)), where θ is the scattering angle. The data were reduced using the SASFLOW pipeline.^21^ 2D SAXS images were radially averaged around the beam center to obtain 1D scattering intensity profiles. For each sample, 40 consecutive frames were acquired and normalized to the transmitted beam, accounting for variations in the sample thickness and beam flux. Background scattering from H_2_O was subtracted to remove the solvent contribution. Final scattering data are presented on an absolute intensity scale calibrated using water as a secondary standard. Resulting scattering curves were analyzed using the free software SASView (http://www.sasview.org/). The add/multiple model tool was used to combine unified_power_Rg model^22–24^ with a hard sphere structure factor.^25,26^ The two-level unified_power_Rg model was used to describe the two levels of cluster organization (clusters and monomers). For both levels, parameters such as power law exponent, B­[level] (amplitude of the structural decay), G (overall scattering power), and radius of gyration (Rg) were left unfixed to be fitted. Both the effective radius (QD’s final radius) and volume fraction corresponding to the structure factor were also kept unfixed. The fitting parameters can be found in section 3.2.1 of the Supporting Information.

## 3 Results and Discussion

### 3.1 Synthesis of Fluorinated Polymers

Fluorinated polymers were prepared following an established protocol of ring opening of the cyclic anhydrides present in commercially available poly­(isobutylene-*alt*-maleic anhydride) (PMA, 6 kDa of average molecular weight) with amino-terminating small molecules,^15^ fluorinated or not (Scheme 1). The composition of the resulting polymers was directly controlled by the stoichiometry used in the reaction between the starting PMA polymer and the amino counterparts and confirmed by ^19^F NMR with an internal standard (TFA) by using a coaxial insert in the NMR tube. Resulting polymers after reaction with fluorinated building block **F** were named **PMA-XF**, where **X** is a number that ranges from 100 to 40 and refers to the percentage of fluorinated monomers in the final polymer (See Table S1 in the Supporting Information). The so-obtained polymers were characterized by NMR (^1^H, ^19^F, and HSQC, see section 2.2 in the Supporting Information). As expected for a polymer, the ^19^F NMR signal obtained consists of a broad band at around −71 ppm, sometimes with other smaller bands nearby, suggesting different environments or arrangements for the polymers when they are in aqueous solution. The potentially unreacted **F** was removed by evaporation, taking advantage of the high volatility of **F**, and further confirmed by the absence of sharp signals in NMR when performed in D_2_O.

**Scheme 1:**
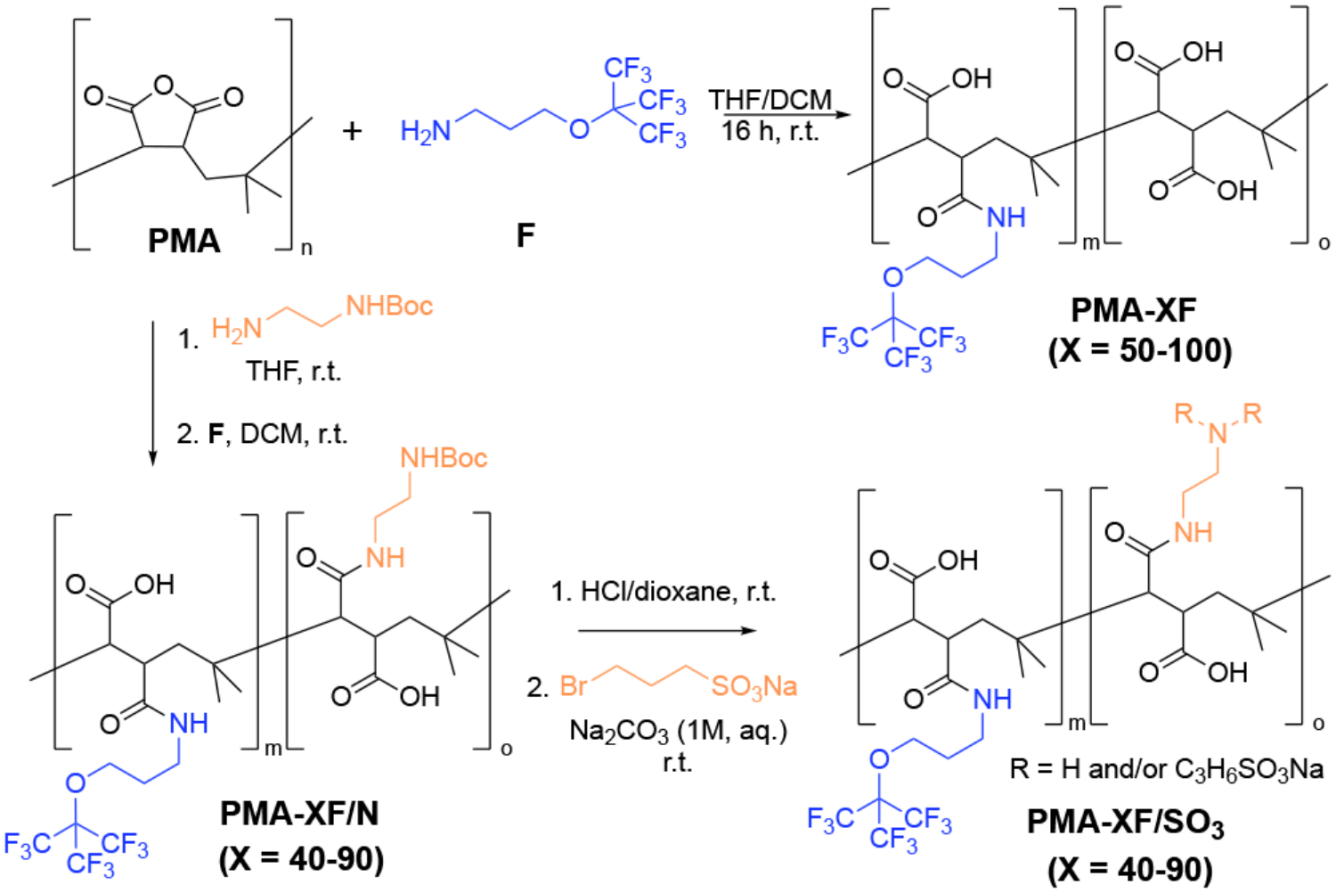
Synthetic Routes for Transforming Commercially Available **PMA** into **PMA-XF** and **PMA-XF/SO**_**3**_ ^a^

a In the case of **PMA-XF**, the number of fluorinated monomers (X) ranged from 50 to 100%, and in the case of **PMA-XF/N** and **PMA-XF/SO**_**3**_, the number of fluorinated monomers (X) ranged from 40 to 90%. A summary of all polymers prepared and the ratio between monomers is shown in Table S1 of the supporting information.

The solubilization in water of these **PMA-XF** polymers requires strong basic conditions (NaOH 0.1–1 M). It must be noted that, despite its effectivity, the use of sodium hydroxide leads to leakage of the fluorinated *tert*-butoxide moiety that takes place very slowly with exposure to a strong base, and it is detected in ^19^F NMR spectra by the appearance of a very sharp, small signal at −75.32 ppm. Although the leaching is very slow (below 2% after 13 days, as measured by ^19^F NMR), we recommend the use of freshly prepared solutions of polymers for immediate use to avoid lack of reproducibility issues that may appear with the use of stored solutions. Nonetheless, after solubilization with a strong base, the basic media may be replaced by water by the use of a desalting column or centrifugal filters of 10 kDa MWCO.

For future applications in which the presence of such a strong base could be a limitation, we also explored the synthesis of partially fluorinated and partially sulfonated polymers that are water-soluble without the need for a strong base and that could be stored in aqueous solution without leaching of fluorine. For this particular case, we first reacted PMA with a single Boc-protected ethylenediamine (10–60% of the PMA monomers). The rest of the monomers were modified with **F**, following the same protocol as in **PMA-XF** polymers. After Boc deprotection with HCl/dioxane, *N*-alkylation with an excess of a bromosulfonate derivative yielded **PMA-XF/SO**_**3**_ polymers (Scheme 1). The modification of only 10% of monomers with sulfonate derivatives already yielded polymers that were soluble in water without the need for a strong base.

### 3.2 Polymer Coating of Gold Nanoparticles

The polymer coating for hydrophobic gold NPs (*d*_c_ = 4.3 ± 0.8 nm) was achieved *via* a very simple phase transfer method. A solution of dodecanethiol-capped gold NPs in dichloromethane was vigorously stirred with a freshly prepared solution of **PMA-XF** in basic solution (NaOH 0.1–1 M) or a stored solution of **PMA-XF/SO**_**3**_ in water. Transfer of the hydrophobic gold NPs from the organic to the water phase took place within the first 30 min of vigorous stirring; however, the mixture was allowed to stir overnight to allow homogeneous coating of the NPs and ensure long-term colloidal stability (Figure 1A). To estimate the amount of polymer needed, we tested different ratios (*R*) of monomers per square nanometer of the surface of the NPs and established that 50 monomers/nm^2^ (R50) was the minimum ratio to ensure colloidal stability. Under these conditions, NPs remained colloidally stable in water for more than 10 days if **PMA-100F** was employed for the polymer coating. However, as soon as the amount of fluorine in the polymer decreased down to 90% and below (**PMA-90F** to **PMA-50F**), we observed some NP precipitation over time (see Figure S8 in the Supporting Information). For these cases, we increased the ratio of polymer monomers per NP surface used for the coating from 50 monomers/nm^2^ (R50) to 75 monomers/nm^2^ (R75), and a long-term colloidal stability was achieved in this manner. The optimal concentration to achieve single NP coating was 0.6 μM for the gold NPs and 3 mM (R50) or 5 mM (R75) in monomers for the fluorinated PMA polymers. We successfully performed the polymer coating of gold NPs using **PMA-50F/SO**_**3**_ (R75) using the same protocol. The so-obtained NPs were characterized by TEM to check that the NPs were coated as single units. The negative staining of TEM samples confirmed that each gold NP was surrounded by a brim of organic polymer independent of the next NP (Figure 1C). This observation was also supported by the UV–vis spectra, which confirmed that the polymer coating did not induce aggregation in solution and that the spectra of the water-dispersed gold NPs with different **PMA-XF** remained unchanged with respect to the original hydrophobic NPs (Figure 1D). The zeta potential analysis (Figure 1E) revealed that the charge decreased from −21.1 ± 0.5 to −31.2 ± 0.2 mV as the fluorine content in the polymer decreased because the number of carboxyl groups is greater as less fluorine is introduced in the polymer. However, for the case of **PMA-50F/SO**_**3**_, we observed a zeta potential of −18.5 ± 1.4 mV, possibly due to the presence of amino groups on the surface of the polymer and the absence of basic media, as these polymers are soluble in plain water.

**Figure 1:**
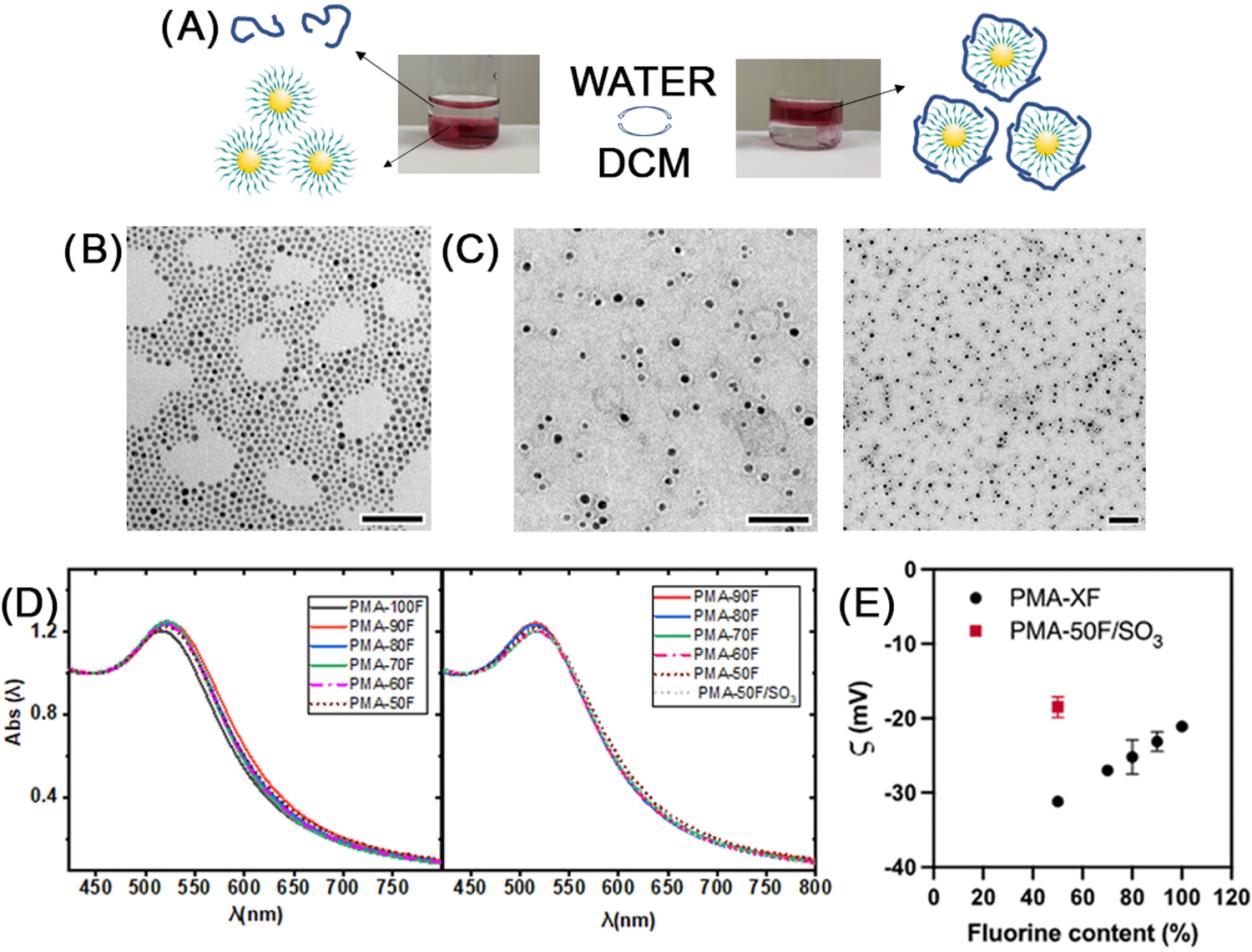
(A) Schematic representation and photos of the phase transfer process for 4 nm gold NPs. The photo on the left shows hydrophobic gold NPs in DCM (bottom red phase), while the transparent top phase contains the **PMA-XF** polymer dissolved in water. The photo on the right shows the polymer-coated gold NPs in the water phase (red top phase) and the transparent bottom phase with DCM and no gold NPs in it. (B) TEM micrograph of dodecanethiol-capped gold NPs before polymer coating (scale bar is 50 nm). (C) TEM micrographs with negative staining at different magnifications of polymer-coated gold NPs with PMA-100F showing single coated NPs (scale bar is 50 nm). (D) UV–vis spectra of gold NPs coated with fluorinated polymers with different fluorine content using a ratio of 50 (left) or 75 (right). (E) Ζ-potential of the polymer-coated NPs.

### 3.3 Polymer Coating of CdSe/ZnS Quantum Dots

The same phase transfer protocol employed for gold NPs was tested on commercially available TOPO-coated CdSe/ZnS QDs; however, despite intense optimization of the polymer to QD ratio and phase dilution, we were unable to transfer the QDs as single NPs, and only polymer-coated aggregates were obtained. To prevent the clustering of QDs and also diminish the contact of QDs with water, thereby preserving their optical properties, we first dispersed both the polymer and QDs in chloroform, dried them slowly, and resuspended the so-obtained film in NaOH (0.1 M) with intense sonication (Figure 2A). A smaller ratio of monomers to QDs of nearly 30 (R28) was used, and careful centrifugation to remove excess of polymer micelles without removing the coating was performed to achieve single encapsulated QDs. The photoluminescence quantum yield (PLQY) of the water-dispersed single QDs was measured for about a week without major changes, thus suggesting that the coating is stable and efficient in preventing water penetration to the core (Figure 2I), which would presumably degrade the nanocrystal and subsequently decrease PLQY.^7^ TEM micrographs (Figure 2C–G) showed a branched fractal pattern that was not observed for gold NPs coated with the same polymers. It is known that fluorine can induce self-assembly due to fluorine–fluorine or hydrophobic interactions.^27^ Although we do not expect to have fluorine accessible on the surface of **PMA-XF**-coated QDs, the denser packing of TOPO (3 alkyl chains per molecule), as compared to single-chain dodecanethiol capping on gold NPs, may force the exposure of some of the bulky fluorinated groups of the polymer for steric reasons, which might interact with other fluorine groups in nearby QDs. Exposure of fluorine to the water phase in fluorinated polymers is reported to happen under specific conditions.^28^ Nonetheless, fractal-like arrangements in polymer-coated NPs are known in the literature.^29,30^ Indeed, QDs present anisotropic, irregular polyhedral-like surfaces that can lead to localized electrostatic repulsion effects, favoring diffusion-limited aggregation. Additionally, the reported TOPO-derived impurities may further disrupt surface passivation, generating heterogeneous regions that act as potential nucleation sites for fractal assembly.^31^

**Figure 2:**
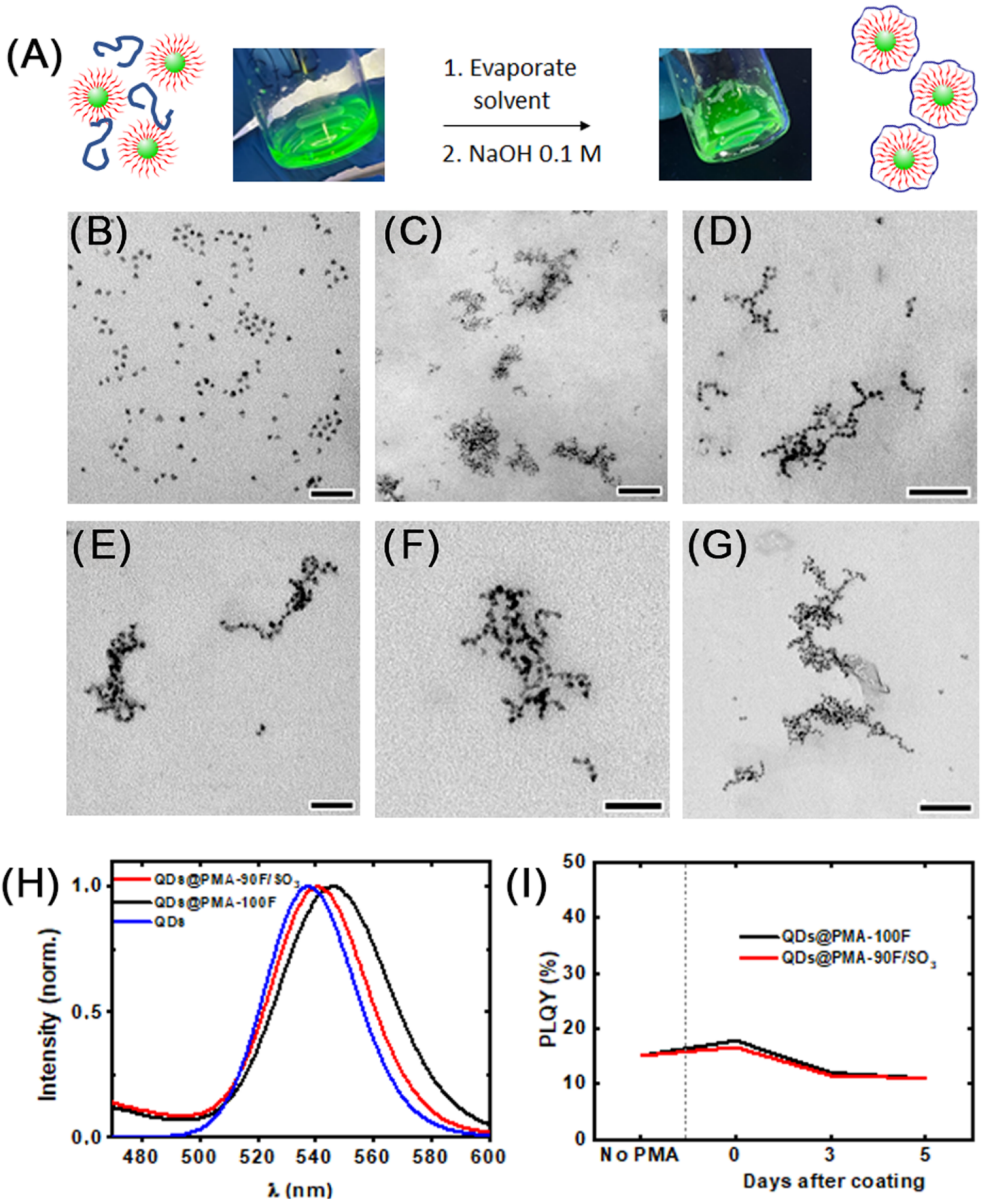
(A) Schematic representation of the polymer coating process with QDs. The photo on the left shows a mixture of polymer and QDs in organic solvent, while the photo on the right shows a water dispersion of polymer-coated QDs. (B) TEM micrograph of commercial TOPO-capped CdSe/ZnS before the polymer coating (scale bar is 50 nm). (C) TEM micrograph of QDs coated with PMA-90F/SO_3_ (scale bar is 100 nm). (D–G) TEM micrographs of QDs coated with PMA-100F showing their arrangement in a branched fractal pattern. Scale bars are 75 nm (D), 50 nm (E and F), and 100 nm (G). (H) Fluorescence spectra of commercial QDs (in CHCl_3_) and polymer-coated ones (in water). (I) Photoluminescence quantum yield (PLQY) evolution over time when using PMA-100F or PMA-90F/SO_3_ as a coating.

It is well-known that the formation of clusters from monomers can be described in terms of fractal aggregates, with the fractal dimension indicating how a fractal fills space. Unlike regular geometric shapes with traditional Euclidean dimensions (e.g., a line with dimension 1 or a square with dimension 2), fractals often have noninteger dimensions that reflect their complex structure, which can be determined by the small-angle X-ray scattering (SAXS) method. SAXS enables the analysis of structural features at the nanoscale directly within the bulk of the liquid sample, eliminating additional manipulations and minimizing the risk of data distortion.^32^ SAXS describes the ultrastructure of the scattering objects, in terms of the outer and inner morphology, in the size range from 1 up to 300 nm according to the expression *d* = 2π/*q*, where *d* is the size of the scattering objects and *q* is the scattering vector. Here, we used SAXS to characterize the structure of coated QDs at two different concentrations (1 and 0.5 μM). Qualitatively, there is a significant difference in the SAXS profiles for the 2 samples at different concentrations, which is associated with the different self-organization of QDs in these systems. We observed differences in the low-*q* (large sizes) scattering region corresponding to scattering from the clusters, the intermediate-*q* region representing monomers, and a peak in the high-*q* (structural correlations of small scale) region, indicating interparticle interactions between the monomers. Such distinctive regions suggest a two-level structural organization, consisting of two coexisting types of QD arrangement, namely, clusters and monomers. Thus, we proceeded to create a scattering model that includes several terms describing these three scattering regions: (1) a unified exponential/power law model to represent the NP core, including a hard sphere structure factor.^25,26^ Another unified exponential/power law model was added to describe the NP clusters.

An important feature of our SAXS data is the change in the slope of the scattering curve as the concentration decreases. Specifically, in the system with a higher concentration of QDs, the power law slope is *p* = 3.3, which corresponds to a surface fractal with a densely packed core and a fractal dimension *D*_s_ = 6 – *p* = 2.7.^32^ In contrast, after dilution, the slope decreases to *p* = 2.3, which corresponds to a highly branched cluster, namely, a mass fractal with a fractal dimension *D*_m_ = *p* = 2.3.^25^ The alignment between the SAXS data and TEM micrographs is remarkable, demonstrating that these NPs form clusters in aqueous solution and these are not artifacts during the sample manipulation for TEM. The formation of large, densely packed clusters at higher concentrations of QD indicates the prevalence of the “monomer-cluster” aggregate growth algorithm in the system, described by Ballistic Limited Aggregation (BLA model), and makes it possible to achieve 1.89 < *D* < 2.95 (Figure 3).^33^

**Figure 3:**
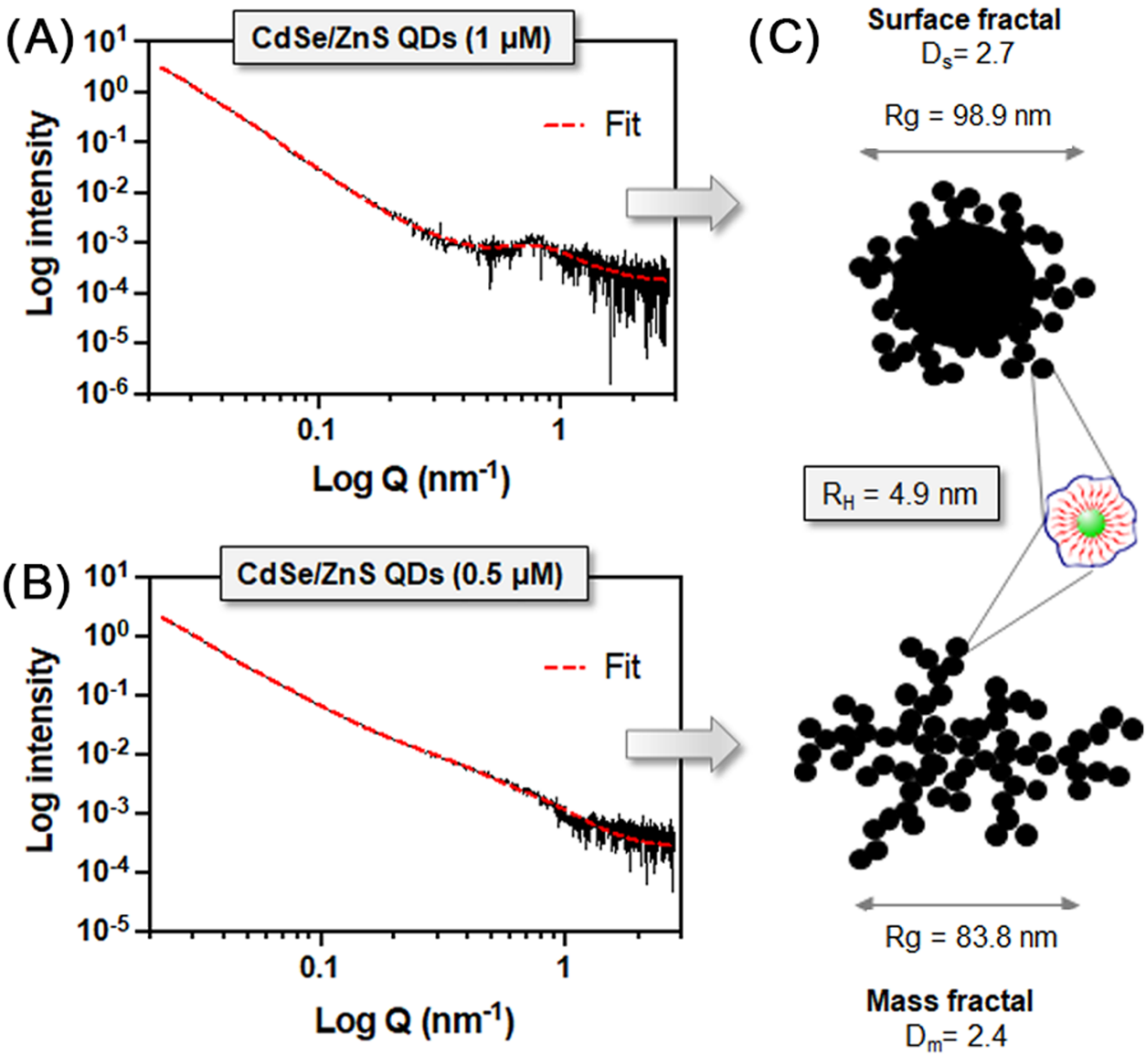
(A) SAXS profile of the CdSe/ZnS QDs coated with PMA-100F showing the features of a surface fractal pattern. (B) SAXS profile of the CdSe/ZnS QDs coated with PMA-100F showing the features of a mass fractal pattern. (C) Scheme and fitted data with the type of fractal obtained at each concentration.

We obtained the radius of gyration for clusters (Rg_cluster_) and monomers (Rg_monomers_) for both concentrated and diluted conditions, assuming a spherical shape of coated QD. Using eq 1 and Rg = 2.3 nm (fitted value), we estimated the coated QD radius in solution to be approximately 4.9 nm. Accounting for the QD core, the shell thickness was estimated to be around 2 nm.

$$radius=\sqrt{\left(\frac{5{R_{g}}^{2}}{3}\right)}$$ 1

Considering the values of the radius of gyration for the clusters, Rg_cluster_ (0.5 μM sample) = 83.8 nm and Rg_cluster_ (1 μM sample) = 98.9 nm, and the previously determined fractal dimension, we calculated the number of quantum dots (*N*) forming a cluster using eq 2.^34^ This resulted in 1666 and 6613 QDs in each cluster for the more diluted and concentrated systems, respectively. Considering the obtained SAXS data, we conclude that an increase in QD concentration leads to an increase in the size of the cluster as well as its packing density.

2 $$N\approx {\left(\frac{{\mathrm{Rg}}_{\mathrm{cluster}}}{{\mathrm{Rg}}_{\mathrm{monomers}}}\right)}^{D}$$

Finally, we calculated the volume fraction of monomers within a fractal cluster using eq 3:

3 $$\varphi \;monomers={\left(\frac{{Rg}_{cluster}}{{Rg}_{monomers}}\right)}^{Df-D}$$

where ϕ is the volume fraction of monomers in the cluster and *D* is the Euclidean dimension of the space (equal to 3 in three-dimensional space). The monomer fraction in the cluster was estimated to be 0.376 (37.6 vol %) and 0.157 (15.7 vol %) for the more concentrated and more diluted systems, respectively. Thus, the volume fraction of solvent (ϕ_solvent_) in both solutions was ϕ_solvent_ = 1–ϕ_monomers_ = 0.624 (62.4 vol %) for concentrated and 0.843 (84.3 vol %) for diluted samples, which means that the more concentrated sample (1 μM) forms more compacted clusters with less content of solvent than the more diluted sample (0.5 μM).

### 3.4 Encapsulation of Highly Fluorinated Molecules in PMA-100F

Apart from NPs, the encapsulation of other hydrophobic cargos was also possible, namely, highly fluorinated compounds such as perfluorocarbon crown-ether (PFCE, MW = 580 Da) and PERFECTA (MW = 408 Da).^19^ Note that these compounds are highly insoluble in basically any known solvent; however, they are interesting as imaging probes for ^19^F MRI due to their high content in equivalent fluorine atoms; hence, their delivery in an aqueous solution is essential for biomedical applications.^35^ The encapsulation methods of such molecules usually require controlled sonication with tips and costly polymers such a PLGA. Highly fluorinated organic molecules such as perfluorocarbons (PFCs) have an affinity for other fluorinated molecules, and sometimes they are only soluble in perfluorinated liquids. Thus, we envisaged that our polymers, having a highly fluorinated side, could interact in a favorable manner with PFCE and PERFECTA to facilitate their encapsulation and transfer to water. PFCE is a liquid that forms a third phase, nonmixable with either water or organic solvents; hence, we encapsulated PFCE in PMA-100F through a simple phase transfer method with the help of a vortex and a benchtop standard sonicating bath. The water phase was analyzed by ^19^F NMR to detect the typical signal of PFCE at −92.86 ppm, usually accompanied by a much smaller peak at −92.65 ppm that was barely detectable depending on the conditions employed (Figure 4A). We were able to encapsulate 30% of the initial PFCE employed under the best conditions (see Table S7 in the Supporting Information). When the same protocol was repeated in the absence of any polymer in the aqueous phase, no signal was detected by ^19^F NMR, thus confirming that the polymer is responsible for transferring insoluble PFCE to the water phase. DLS analysis (Figure S18 and Table S8 in the Supporting Information) revealed the formation of NPs of 211.9 ± 11.1 nm with a polydispersity index (PdI) of 0.23. With the aim to reduce the PdI and homogenize the samples, we filtered them through 0.45 μm syringe filters prior to DLS measurements. The DLS data remained quite similar, affording a size of 192.1 ± 4.2 nm and a PdI of 0.18, but a substantial amount of sample was lost in the filters, and it also led to an increase in the signal intensity of the peak at −92.65 ppm in ^19^F NMR, suggesting a change in how the PFCE and polymer are arranged and interact with one another (Figure 4C).

**Figure 4:**
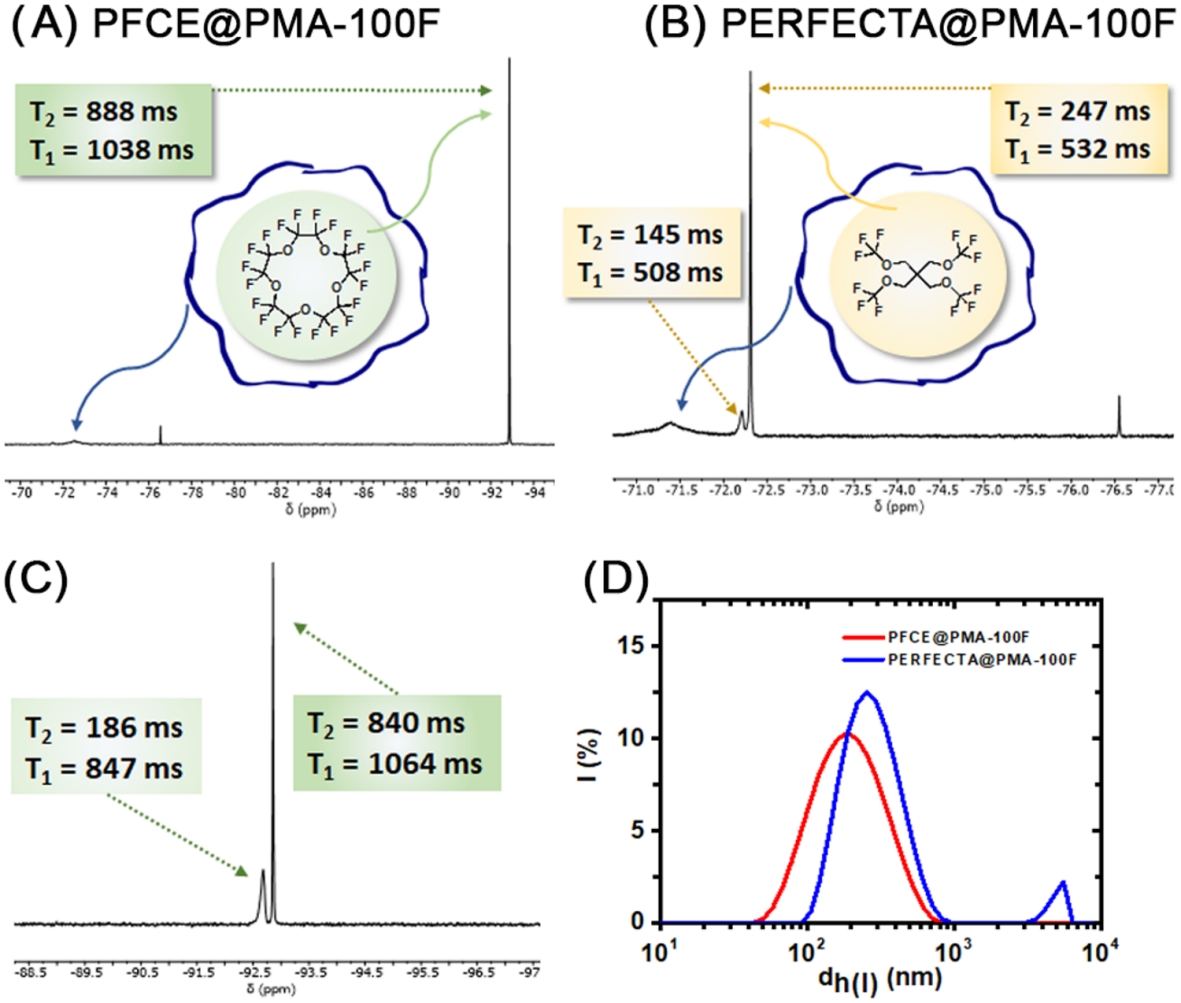
(A) ^19^F NMR spectrum of encapsulated PFCE showing the corresponding sharp signal at around −93 ppm and the broad bands from the polymer at −71 ppm. TFA (−76.55 ppm) was used as a reference. (B) ^19^F NMR spectrum of encapsulated PERFECTA showing the corresponding PERFECTA signals at around −72 ppm, and the broad bands from the polymer at −71 ppm. TFA (−76.55 ppm) was used as a reference. (C) ^19^F NMR spectrum of encapsulated PFCE after filtration showing the corresponding sharp signal at around −93 ppm and a broader signal at −92.65 ppm. (D) DLS results for PFCE or PERFECTA encapsulated in **PMA-100F**.

For PERFECTA, being a solid, we followed a different protocol in which we mixed it with **PMA-100F** in chloroform under reflux overnight and then evaporated the solvent, and the so-obtained film was reconstituted in NaOH (0.1 M). The aqueous solution was analyzed by ^19^F NMR to detect PERFECTA signals, and again a characteristic sharp peak at −72.3 ppm was detected together with a broader signal at −72.2 ppm (Figure 4B). With this method, we were able to transfer to water 80% of the initial PERFECTA added (see Table S10 in the Supporting Information). A blank experiment in which **PMA-100F** was not added resulted in a nondetectable PERFECTA signal in ^19^F NMR in the aqueous phase. The encapsulated PERFECTA was transferred with centrifugal filters of 10 kDa MWCO to either water or NaHCO_3_ 0.1 M, and the colloidal stability was studied over a week by DLS. Minimal changes in size were observed over time, though the samples were rather polydisperse (PdI between 0.25 and 0.44) (see Figure S24 and Table S11 in the Supporting Information). The most interesting applications of PFCE and PERFECTA are in the field of ^19^F MRI, and thus, the transverse (*T*_2_) and longitudinal (*T*_1_) relaxation times of **PMA-100F** encapsulated PFCE and PERFECTA were measured. PFCE encapsulated NPs afforded very high *T*_2_ values of 888 ms, and reasonable *T*_1_ values of 1030 ms (see Table S9 and Figure S19 in the Supporting Information). High *T*_2_ values are interesting for the design of probes for ^19^F MRI because they lead to a high signal-to-noise ratio, and a value of 888 ms is well above reported *T*_2_ values for PFCE encapsulated in other polymers, which usually range from 300 to 500 ms.^36^ After filtration, the sharp peak at −92.86 ppm delivered similar relaxation times (*T*_2_ = 840 ms and *T*_1_ = 1064 ms), while the broad peak at −92.65 ppm showed a much shorter *T*_2_ of 186 ms and a similar *T*_1_ of 847 ms, which suggests that there is a population of fluorine atoms in the PFCE core that have reduced mobility, perhaps those in closer contact with the surrounding polymer (see Table S9 and Figure S20 in the Supporting Information). Likewise, the relaxation times for PERFECTA were *T*_2_ = 247–153 ms and *T*_1_ = 595–519 ms for the signal at −72.3 ppm; or *T*_2_ = 145–88 ms and *T*_1_ = 566–508 ms for the broader signal at −72.2 ppm (see Table S12, Figures S25 and S26 in the Supporting Information). The presence of fluorine atoms with substantially different *T*_2_ values, particularly for PFCE, suggests a spatial arrangement in which those nuclei with shorter *T*_2_ values have clearly less mobility than those with higher *T*_2_ values. Different spatial arrangements for these fluorinated molecules in PLGA polymers have indeed been reported before.^37^

As mentioned before, to the best of our knowledge, this is the first time that fluorinated polymers have been employed to encapsulate hydrophobic NPs such as gold NPs and QDs and transfer them to water. With respect to ^19^F MRI probes, a fluorinated polymer has previously been described as an efficient transfer-to-water agent for PERFECTA.^38^ However, that polymer is based on a perfluorocarbon chain, which exhibits significantly greater resistance to degradation and therefore higher persistence compared to the smaller perfluoro *tert*-butyl ether we employ. Our compound is susceptible to degradation under basic conditions, and similar ether bonds are known to undergo oxidative degradation in biological environments.^39^ Additionally, we already reported the use of the same perfluoro *tert*-butyl ether moiety on PEG polymers and onto gold NPs with no evidence of cytotoxic effects in four different cell lines^40,41^ or after their *in vivo* administration.^16^ In this context, our novel polymers represent a significant advance in the transfer of hydrophobic cargos. Compared to other materials, these polymers are exceptionally easy to synthesize, and we have demonstrated that they can be easily modified owing to the anhydride moiety of commercially available PMA, which could react with a wide variety of nucleophiles (e.g., complex amines and alcohols). This makes polymer modification straightforward, even avoiding the use of prefunctionalized moieties, since the chemical modification onto the polymer is feasible. Furthermore, our polymer is highly versatile, showing outstanding performance in transferring either various nanocrystals or discrete organic molecules to water. Finally, in comparison with other PMA-derived polymers bearing nonfluorinated hydrophobic chains,^15,42,43^ our polymers present a comparable performance while providing a more sophisticated platform that potentially allows the coating and transfer to water of extremely hydrophobic materials into aqueous environments.

## 4 Conclusions

In this work, we showed that fluorinated amphiphilic polymers can be prepared very easily by ring opening of the anhydrides present in a commercially available PMA polymer. The content of fluorine in the resulting polymers can be controlled by the stoichiometry of the reaction, and it can be combined with other functional groups. Those polymers take advantage of the intrinsic hydrophobicity of fluorinated derivatives and enable the encapsulation of single NPs (gold NPs and CdSe/ZnS QDs) that remained colloidally stable in solution. For the particular case of QDs, their optical properties were preserved in the process of being transferred to water for at least 5 days. In addition to NPs, also perfluorinated PFCE or almost perfluorinated PERFECTA compounds with interesting applications as probes for ^19^F MRI were encapsulated. While the encapsulation efficiency of PFCE was not particularly high (30%), the transverse relaxation times obtained were higher than others reported for the same compound wrapped in other polymers. On the contrary, the encapsulation efficiency of PERFECTA was much higher (80%), although the DLS shows a polydisperse sample, and the relaxation times are similar to reported values with other polymers. Hence, we conclude that fluorinated polymers can be used for encapsulation purposes of both NPs or hydrophobic compounds and may be particularly useful for cargos that need to avoid contact with water to preserve their properties.

## Supplementary Material



## Notes

Additional experimental data and detailed characterization data, including 1D and 2D NMR spectra, TEM micrographs, DLS, relaxation times measurements, and SAXS fitting data (PDF)
