# Supplementary material for: Synthesis of Novel Amphiphilic Fluorinated Polymers for the Dispersion of Hydrophobic Gold Nanoparticles, Quantum Dots, or Highly Fluorinated Molecules in Water
Source: ACS Nanosci Au. 2025 Sep 27;5(6):482–92. doi: 10.1021/acsnanoscienceau.5c00069 (PMC12715620; doi:10.1021/acsnanoscienceau.5c00069)
Supplement: Supplementary file 1 [file ng5c00069_si_001.pdf]

Synthesis of novel amphiphilic fluorinated polymers for the dispersion of hydrophobic gold nanoparticles, quantum dots or highly fluorinated molecules in water.

Galder Llorente,[a*] Juan Manuel Arango,[b,c*] Noelia Soto,[b,d] Olena Kyzyma,[b] Andres Alejandro Yanez Crespo,[b] Clement Blanchet,[e] Aitzol Garcia-Etxarri,[a,f] Marite Cardenas,[b,d,f] Monica Carril.[b,c,f‡]

** Both authors contributed equally*

‡ E-mail: monica.carrilg@ehu.eus

[a] *Donostia International Physics Center, Paseo Manuel de Lardizabal 4, 20018, Donostia-San Sebastian, Spain*

[b] *Instituto Biofisika (UPV/EHU, CSIC), University of the Basque Country, Barrio Sarriena s/n, 48940, Leioa, Spain.*

[c] *Departamento de Bioquímica y Biología Molecular, University of the Basque Country (UPV/EHU) Barrio Sarriena s/n, 48940, Leioa, Spain.*

[d] *Fundación Biofísica Bizkaia/Biofisika Bizkaia Fundazioa (FBB), Barrio Sarriena s/n, 48940, Leioa, Spain.*

[e] *European Molecular Biology Laboratory EMBL, Hamburg Unit, c/o DESY Notkestrasse 85, 22603, Hamburg, Germany.*

[f] *IKERBASQUE, Basque Foundation for Science, Plaza Euskadi 5, 48009, Bilbao, Spain.*

INDEX

1. General remarks 3

2. Fluorinated PMA polymers 4

2.1. Synthesis of fluorinated PMA polymers 4

2.2. ^{1}H, ^{19}F and 2D HSQC NMR spectra of fluorinated polymers 4

3. Polymer coating of nanoparticles 10

3.1. Characterisation of polymer coated gold NPs 10

3.2. Characterisation of polymer coated CdSe/ZnS QDs 11

3.2.1. SAXS fitting parameters 12

4. Encapsulation and water transfer of PFCE 14

4.1. ^{19}F-NMR spectra of PMA-100F coated PFCE 14

4.2. DLS analysis of PMA-100F coated PFCE 18

4.3. T_2 and T_1 relaxation times of fluorine 19

5. Encapsulation and water transfer of PERFECTA 21

5.1. Synthesis of PERFECTA 22

5.2. ^{19}F-NMR of PERFECTA encapsulated in PMA-100F in NaOH 0.1 M and after transfer to water or $NaHCO_3$ 0.1 M 23

5.3. DLS analysis of PMA-100F coated PERFECTA 24

5.4. T_1 and T_2 relaxation times of PERFECTA in PMA-100F 25

1. General remarks

All reagents were purchased and used as received. All air- or moisture-sensitive reactions were performed under nitrogen atmosphere. Fluorinated building block **F** was prepared as described previously by us[1] following the synthetic route depicted in Scheme S1. ^{1}H NMR, ^{13}C NMR and ^{19}F spectra were recorded on a Bruker AC-300 instrument (300 MHz for ^{1}H, 75.4 MHz for ^{13}C and 283 MHz for ^{19}F) at 20 °C. 2D NMR spectra were acquired on a Bruker AC 500 instrument (500 MHz for ^{1}H and 125.7 MHz for ^{13}C). Chemical shifts (δ) are given in ppm downfield from Me_4Si and are referenced as internal standard to the residual solvent $CDCl_3$ (δ = 7.26 for ^{1}H and δ = 77.16 for ^{13}C), except for ^{19}F NMR in which trifluoroacetic acid (TFA) was used as a reference (δ = -76.55 ppm). TLC was carried out on SiO_2 (silica gel 60 F254, Merck), and the spots were located with UV light or using different staining solutions if needed. Flash chromatography was carried out on a Büchi C-185 automated flash chromatography system or by conventional flash chromatography on SiO_2 (silica gel 60, Merck, 230–400 mesh ASTM). Drying of organic extracts during work-up of reactions was performed over anhydrous Na_2SO_4. Evaporation of solvents was accomplished with a Büchi rotatory evaporator. HRMS spectra were recorded on a HPLC Vanquish coupled to an Orbitrap Exploris MX spectrometer by electrospray ionization (ESI).

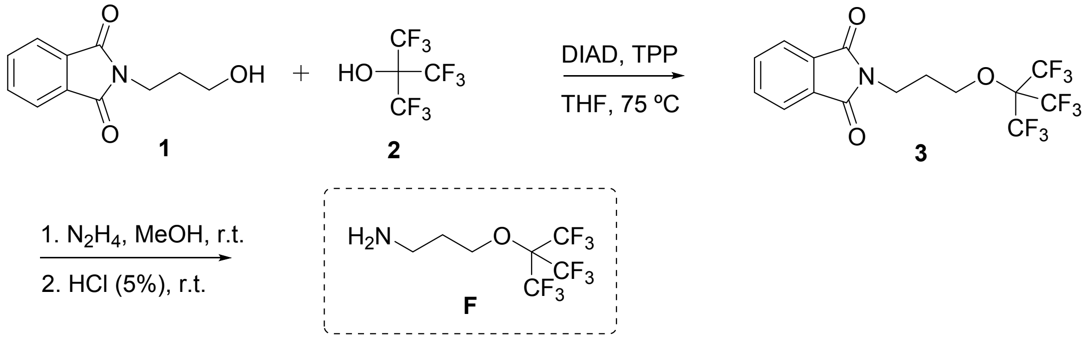

*Scheme S1. Synthesis of building block **F**.*

[1] Arango, J. M.; Padro, D.; Blanco, J.; Lopez-Fernandez, S.; Castellnou, P.; Villa-Valverde, P.; Ruiz-Cabello, J.; Martin, A. Fluorine Labeling of Nanoparticles and In Vivo ^{19}F Magnetic Resonance Imaging. *ACS Appl. Mater. Interfaces* **2021**, *13*, 12941–12949.

2. Fluorinated PMA polymers

2.1. Synthesis of fluorinated PMA polymers

A family of fluorinated polymers with different fluorine content were synthesized. The fluorination reaction takes place after a simple ring opening reaction of amino ending **F** on a commercially available polymer poly-isobutylene-*alt*-maleic anhydride (**PMA**). According to the manufacturer, PMA has a molecular weight of approximately 6000 Da. Considering the average molecular weight of the monomeric unit, which is 154.046 Da (specified by the provider), the approximate number of monomeric units (n) was calculated to be 39. The fluorination degree on PMA is controlled by the stoichiometry of the reaction and was calculated from ^{19}F NMR (δ = -71.90 ppm) signal integration using TFA as reference compound. A summary of the prepared fluorinated polymers and the degree of fluorination is shown in Table S1.

*Table S1. Amount of **F** used in the synthesis of each polymer **PMA-F***

PMA-F sample	F (eq.)	H_2N-C_2H_4-NHBoc (eq.)	Degree of fluorination as detected by ^{19}F NMR (%)
**PMA-50F**	0.5	0	49 %
**PMA-60F**	0.6	0	60 %
**PMA-70F**	0.7	0	71 %
**PMA-80F**	0.8	0	79 %
**PMA-90F**	0.9	0	90 %
**PMA-100F**	1.1	0	100 %
**PMA-90F/SO_3**	1	0.1	92 %
**PMA-60F/SO_3**	0.6	0.4	65 %
**PMA-50F/SO_3**	0.5	0.5	52 %
**PMA-40F/SO_3**	0.4	0.6	41 %

2.2. ^{1}H, ^{19}F and 2D HSQC NMR spectra of fluorinated polymers

For ^{19}F-NMR measurements, the polymer was either dissolved in NaOH 0.1 M or in a mixture of THF and MeOD. A coaxial insert loaded with TFA (0.077 % v/v) in deuterium oxide was used as an internal standard. When using only organic solvents, the ^{19}F NMR

signals obtained are sharper than when in water solution. For ^{1}H-NMR (watergate) and 2D HSQC, the sample was dissolved in NaOH 0.1 M and the experiment was performed with a coaxial insert loaded with deuterium oxide for shimming.

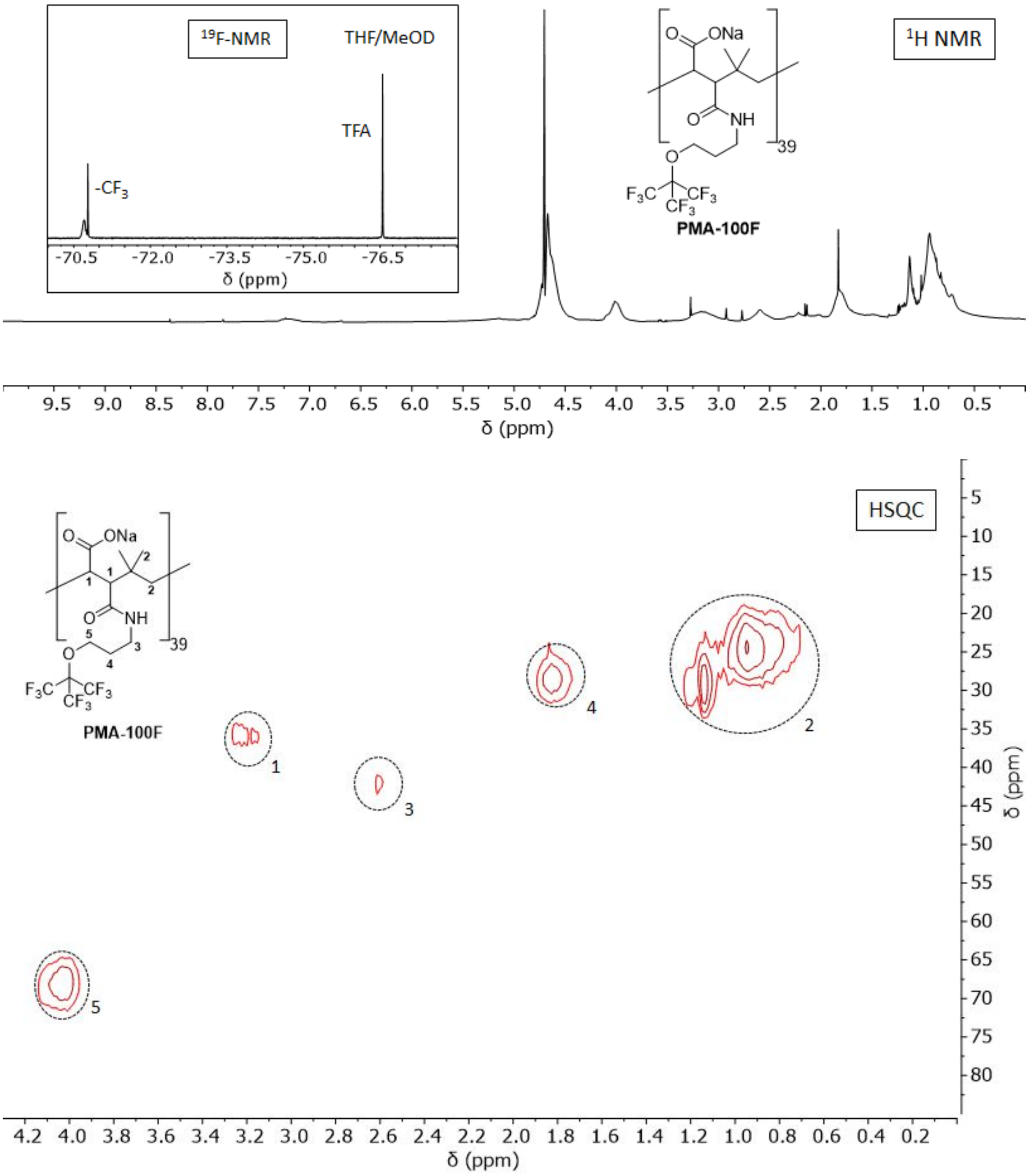

*Figure S1. ^{1}H-NMR (top) and HSQC (bottom) spectra of **PMA-100F**.*

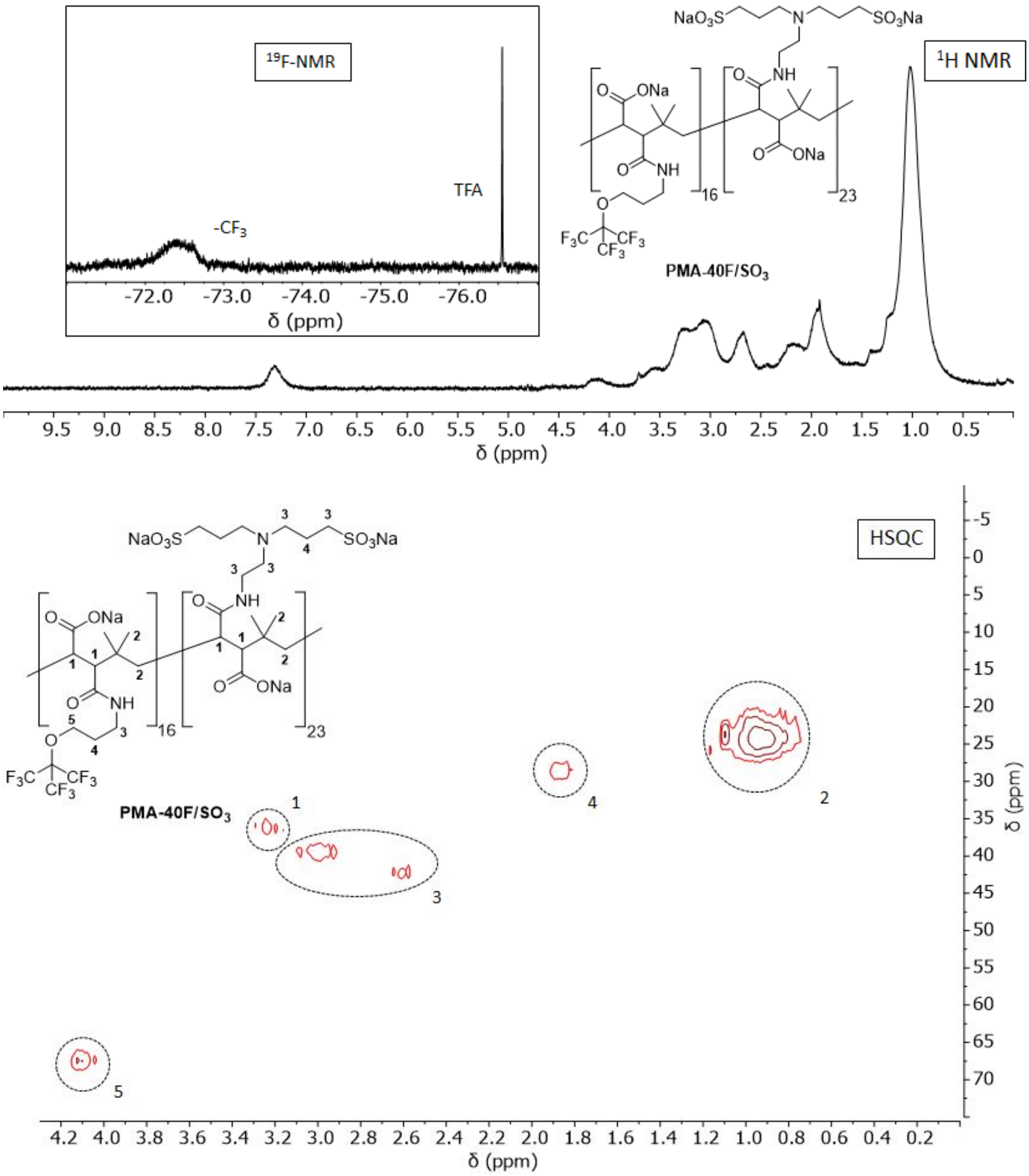

*Figure S2. ¹H-NMR (top) and HSQC (bottom) spectra of **PMA-40F/SO₃**.*

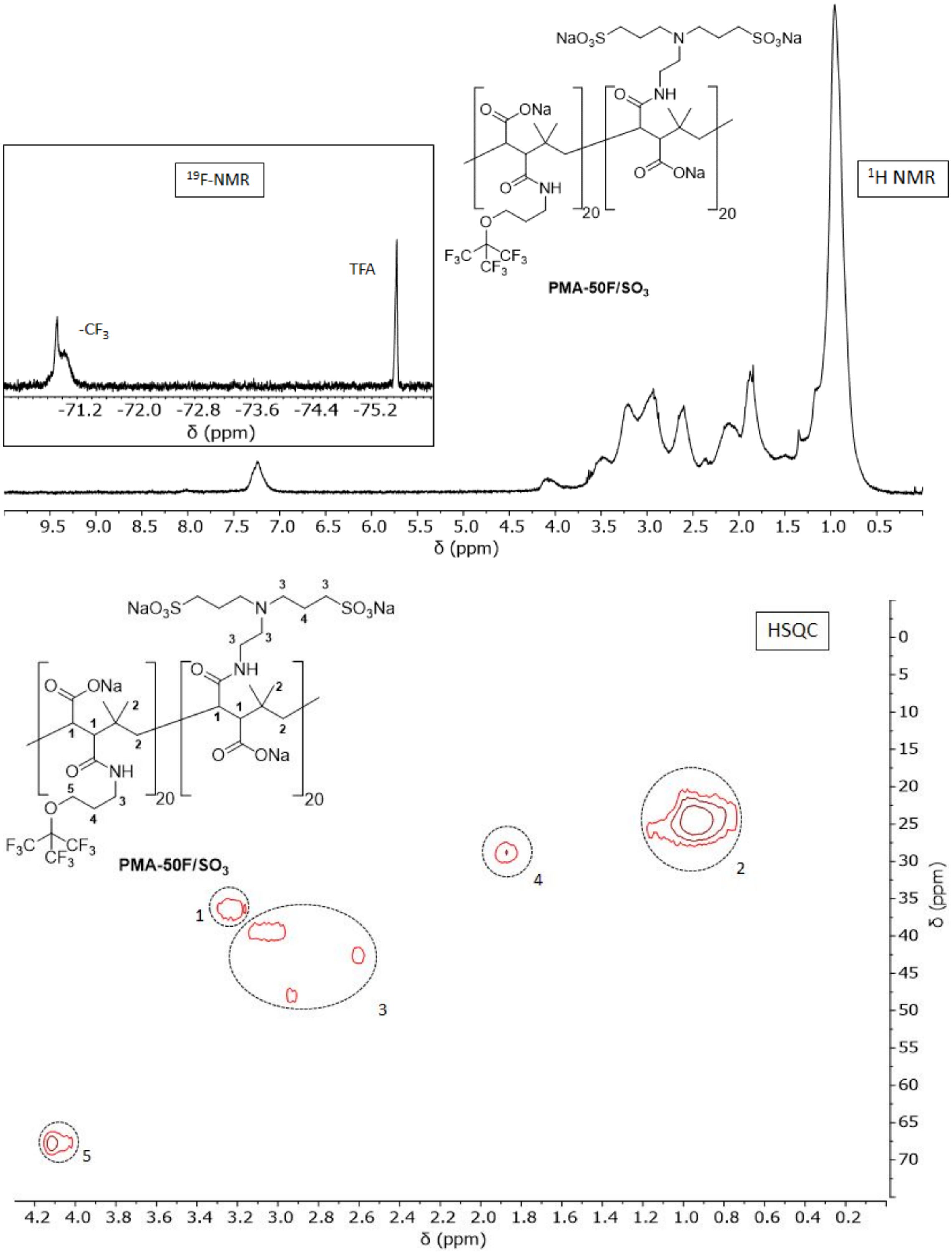

*Figure S3. ^{1}H-NMR (top) and HSQC (bottom) spectra of **PMA-50F/SO$_3$**.*

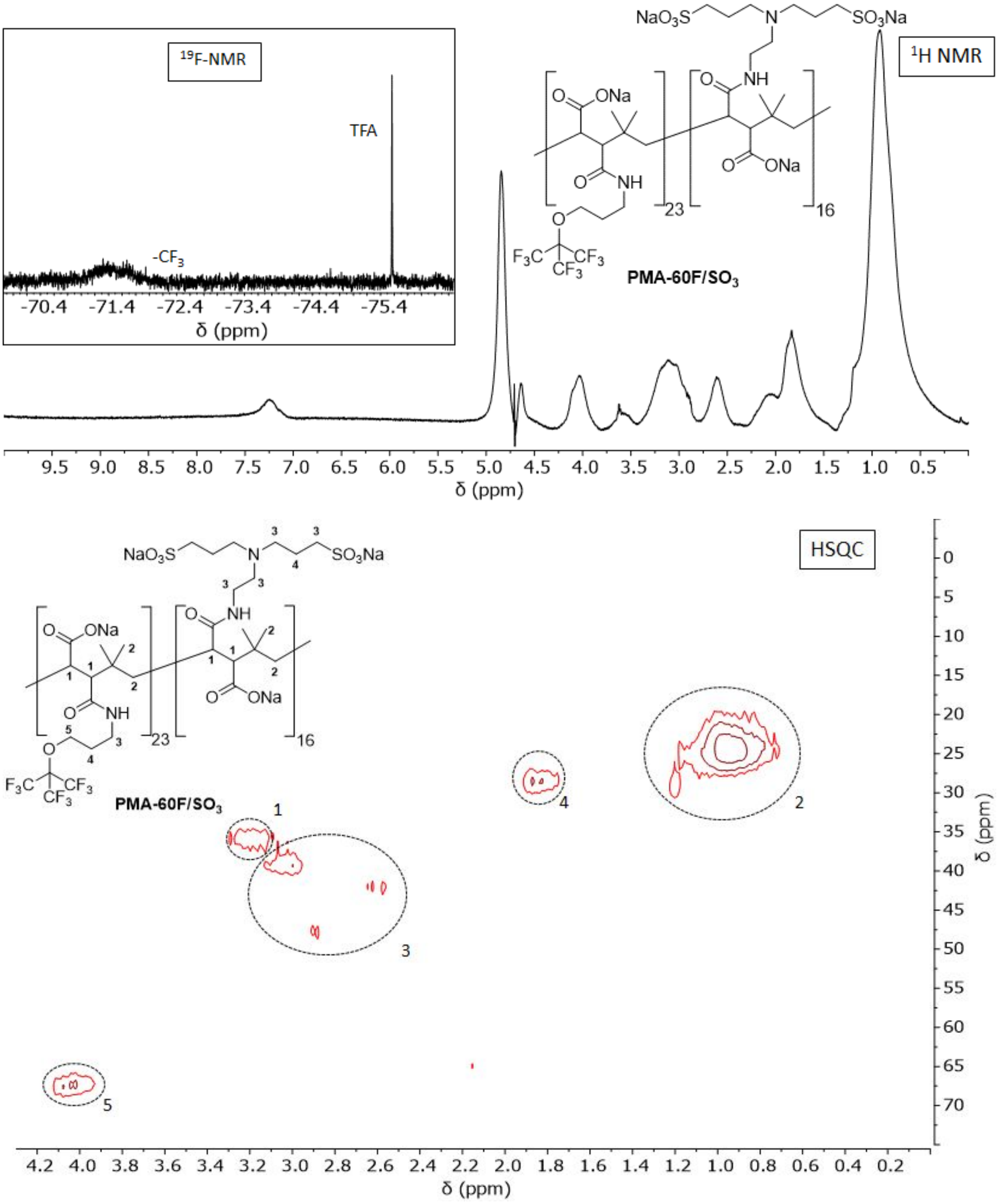

*Figure S4. ^{1}H-NMR (top) and HSQC (bottom) spectra of **PMA-60F/SO$_3$**.*

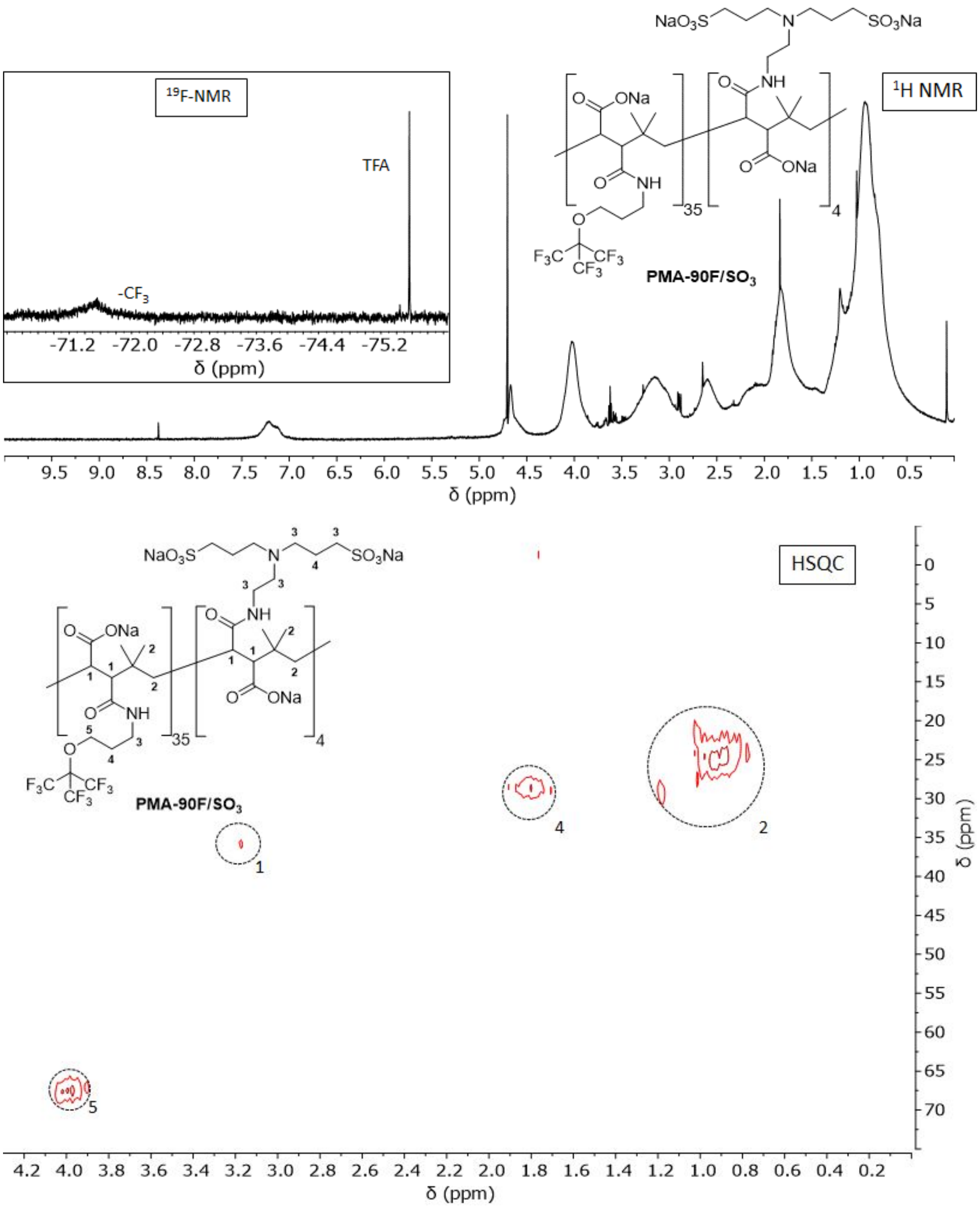

*Figure S5. ^{1}H-NMR (top) and HSQC (bottom) spectra of **PMA-90F/SO₃**.*

3. Polymer coating of nanoparticles

3.1. Characterisation of polymer coated gold NPs

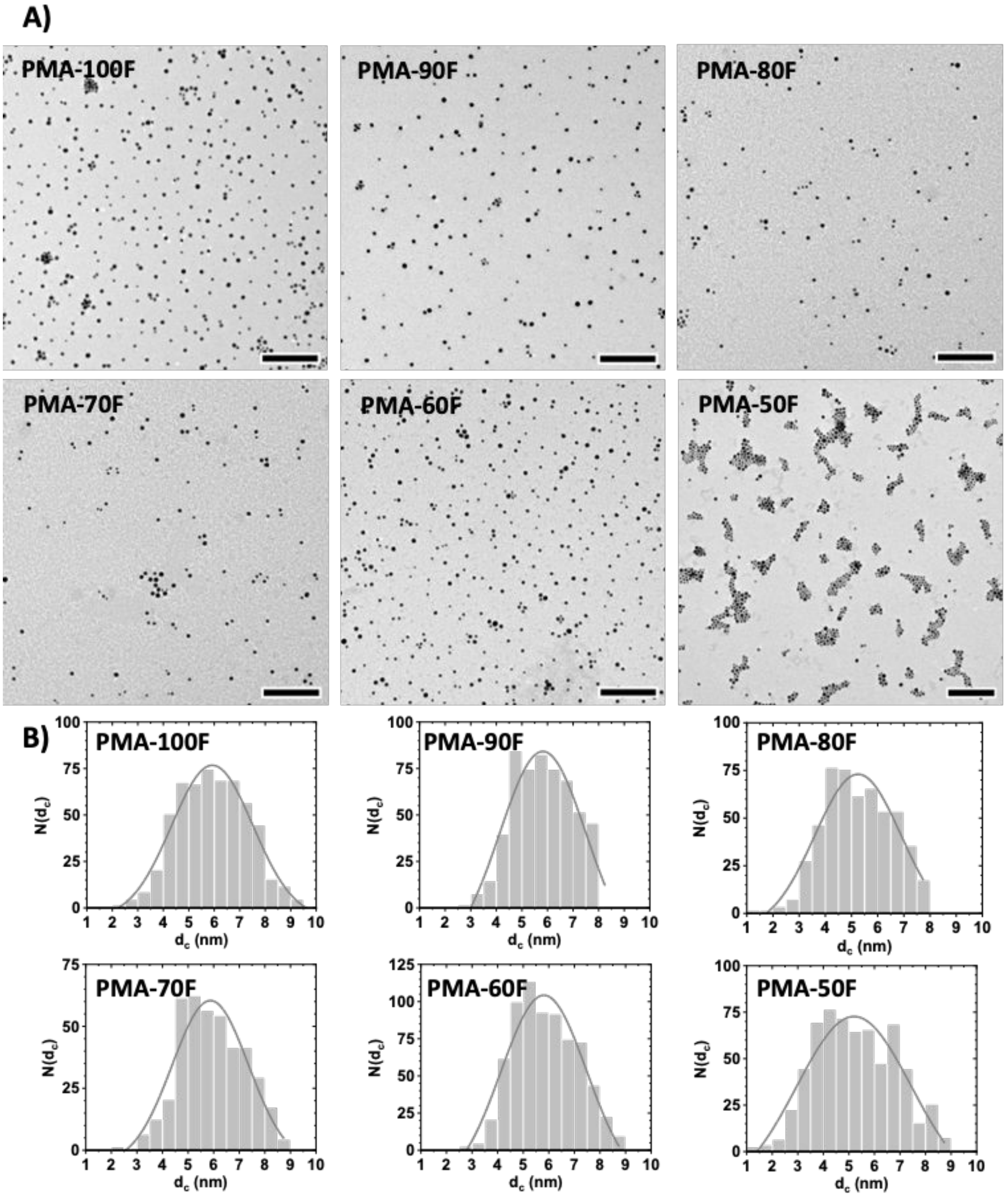

Figure S6. TEM micrographs (A) and the size histogram (B) for each PMA-XF and gold NPs using a ratio of 50 (R50). All scale bars are 100 nm.

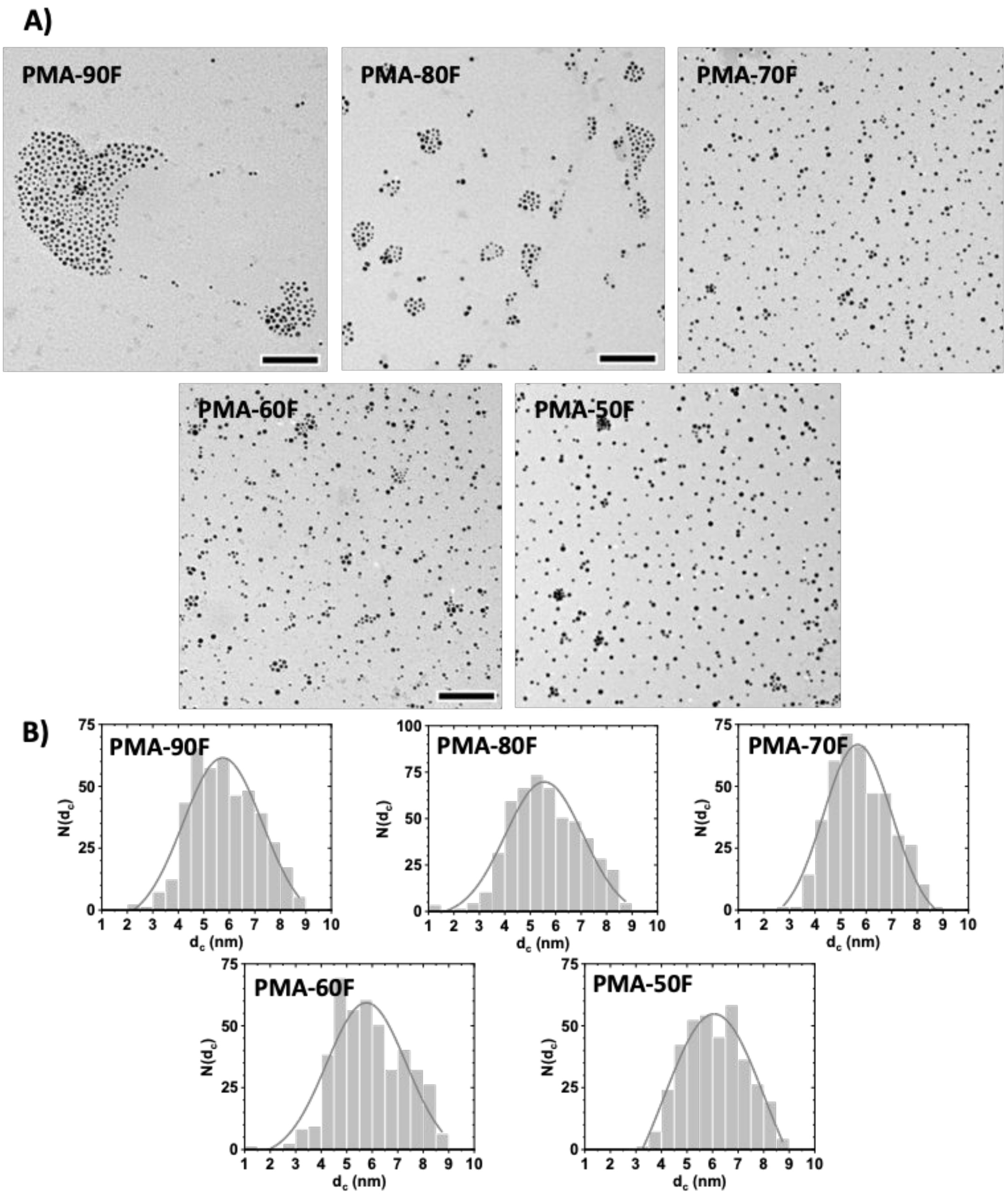

Figure S7. TEM micrographs (A) and the size histogram (B) for each PMA-XF and gold NPs using a ratio of 75 (R75). All scale bars are 100 nm.

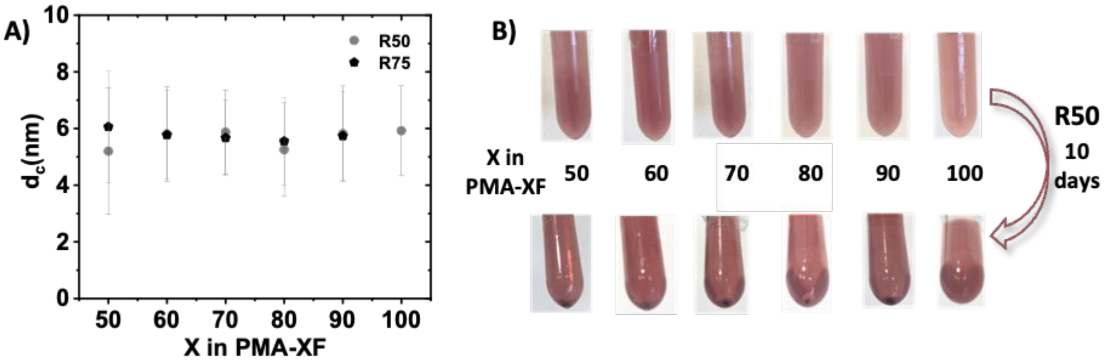

Figure S8. A) Summary of the size obtained for each coating experiment shown in Figures S6 and S7. B) Photographs showing precipitation of NPs after 10 days when using R50 and polymers with 90 % of fluorine or less. The only sample without precipitate is that of PMA-100F. The colloidal stability for those polymer coated NPs was improved by using R75.

Table S2. Core diameter for each coating experiment as obtained from TEM image analysis.

PMA-XF	d_c (nm) (R50)	d_c (nm) (R75)
**PMA-100F**	5.9 ± 1.6	-
**PMA-90F**	5.8 ± 1.7	5.7 ± 1.6
**PMA-80F**	5.2 ± 1.6	5.5 ± 1.5
**PMA-70F**	5.9 ± 1.5	5.7 ± 1.3
**PMA-60F**	5.8 ± 1.7	5.8 ± 1.6
**PMA-50F**	5.2 ± 2.2	6.1 ± 1.9

3.2. Characterisation of polymer coated CdSe/ZnS QDs

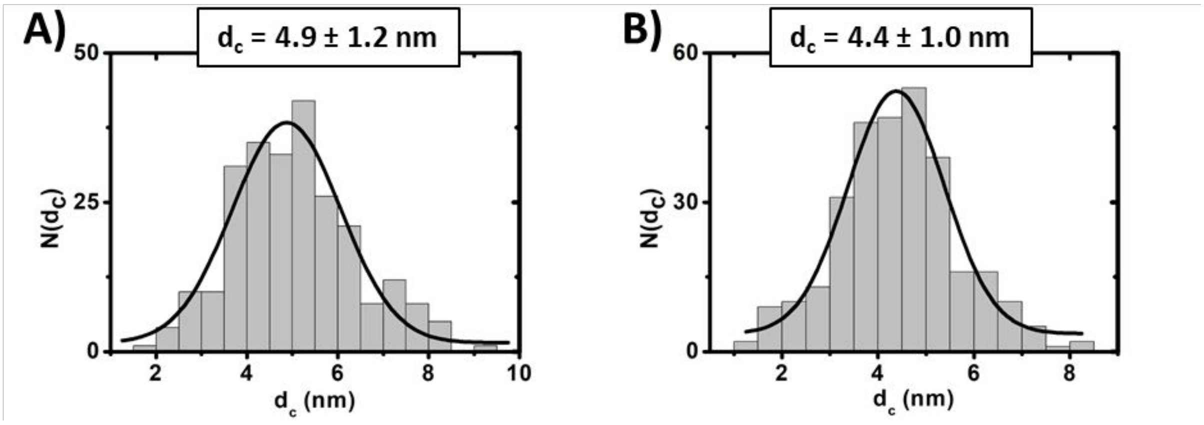

Figure S9. Size histograms and gaussian fit of A) PMA-100F coated QDs and B) PMA-90F/SO_3 coated QDs.

Table S3. Summary of particle diameter, PLQY, Em. λ_{max} and FWHM for each sample.

NP sample	d_c (nm)	PLQY (%, solvent)	Em. λ_{max} (nm, solvent)	FWHM (nm)
**TOPO-capped QDs**	6.2 ± 1.0	15.1 ($CHCl_3$)	538 ($CHCl_3$)	36
**QDs@PMA-100F**	4.9 ± 1.2	17.8 (NaOH 0.1 M)	546 (NaOH 0.1 M)	44
**QDs@PMA-90F/SO_3**	4.4 ± 1.0	16.6 (H_2O)	541 (H_2O)	39

A small value for the Full Width at Half Maximum (FWHM) of the fluorescence emission is a sign of the quality of the QD. This parameter is usually increased with QDs are

transferred to water and it is an indication of loss of optical properties. We observed that when using **PMA-90F/SO$_3$** the FWHM remains very similar to that of the original QDs in organic solvent, but it is slightly increased with using **PMA-100F**.

Table S4. PLQY measurements at different days of A) polymer coated QDs using PMA-100F (in NaOH 0.1 M) and B) polymer coated QDs using PMA-90F/SO$_3$ (in H_2O).

NP sample	PLQY (%, day 0)	PLQY (%, day 3)	PLQY (%, day 5)
**QDs@PMA-100F**	17.83	12.02	10.98
**QDs@PMA-90F/SO$_3$**	16.61	11.47	10.97

3.2.1. SAXS fitting parameters

Table S5. Fitting parameters for 1 µM QDs solution.

**1 µM QDs solution**			
**Parameter**	**Value**	**Error**	**Units**
**Background**	5.0009×10^{-5}	$\pm 1.0213 \times 10^{-5}$	cm^{-1}
**A_scale**	0.0090888	± 0.00012311	—
**B_scale**	0.00011906	$\pm 1.0322 \times 10^{-5}$	—
**radius_effective**	3.813	± 0.031736	nm
**Volfraction**	0.19632	± 0.0061322	—
**$Rg_{cluster}$**	98.868	± 1.0693	nm
**Power law $exp_{cluster}$**	3.3019	± 0.0069071	—
**$B_{cluster}$**	0.0016304	$\pm 3.3286 \times 10^{-5}$	cm^{-1}
**$G_{cluster}$**	1466.6	± 39.376	cm^{-1}
**$Rg_{monomer}$**	3.813	± 0.031736	nm
**Power law $exp_{monomer}$**	3.8102	± 0.01507	—
**$B_{monomer}$**	0.073141	± 0.0024079	cm^{-1}
**$G_{monomer}$**	0.14793	± 0.0060195	cm^{-1}

Table S6. Fitting parameters for 0.5 µM QDs solution

0.5 µM QDs solution			
**Parameter**	**Value**	**Error**	**Units**
**Background**	0.00016461	— (fixed)	cm^{-1}
**A_scale**	0.00018595	$\pm 2.192 \times 10^{-6}$	—
**B_scale**	9.7206×10^{-5}	$\pm 2.8193 \times 10^{-6}$	—
**radius_effective**	3.813	± 0.031736	nm
**Volfraction**	0.13083	± 0.032111	—
**$Rg_{cluster}$**	83.801	± 0.51391	nm
**Power law $exp_{cluster}$**	2.385	± 0.007226	—
**$B_{cluster}$**	1.3142	± 0.028117	cm^{-1}
**$G_{cluster}$**	31127	± 318.23	cm^{-1}
**$Rg_{monomer}$**	3.813	± 0.031736	nm
**Power law $exp_{monomer}$**	3.8102	± 0.01507	—
**$B_{monomer}$**	5.9766	± 0.13422	cm^{-1}
**$G_{monomer}$**	53.277	± 0.98336	cm^{-1}

4. Encapsulation and water transfer of PFCE

Table S7. Added amount of PFCE to be encapsulated and amount of PFCE detected in water by ^{19}F NMR using a TFA internal standard.

Sample	PFCE added (µmol)	PFCE in H_2O (µmol)	Encapsulation efficiency (%)	Sample-Filt	Encapsulation efficiency (%)
**PFCE-1**	15.35	4.58	30	**PFCE-1-Filt**	5
**PFCE-2**	30.96	3.17	10	**PFCE-2-Filt**	3
**PFCE-3**	92.07	3.49	4	**PFCE-3-Filt**	<1
**PFCE-4**	153.50	1.13	<1	**PFCE-4-Filt**	<1

The encapsulation efficiency corresponds to the percentage of initial PFCE that has been transferred to water. Sample-Filt refers to samples obtained after passing through a 0.45 µm syringe filter. The filtering step induces loss of material as it can be seen in the encapsulation efficiency of the filtered samples but also affects the ^{19}F NMR signal and relaxation times.

4.1. ^{19}F-NMR spectra of PMA-100F coated PFCE

For ^{19}F-NMR measurements, the sample was dissolved in NaOH 0.1 M and the experiments were performed with a coaxial insert loaded with TFA (0.024 % v/v) in deuterium oxide).

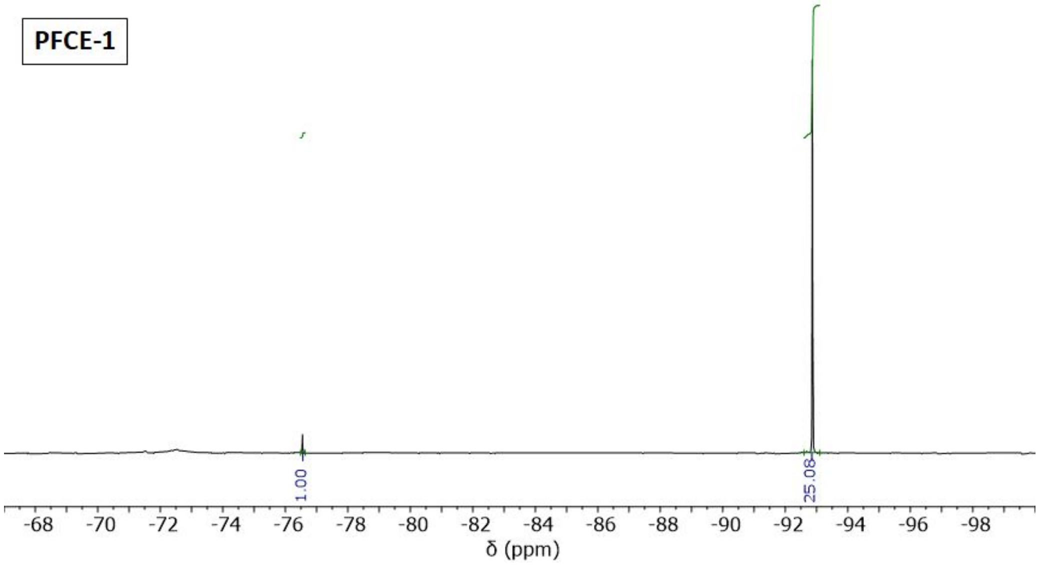

*Figure S10. ^{19}F-NMR spectra of **PFCE-1**.*

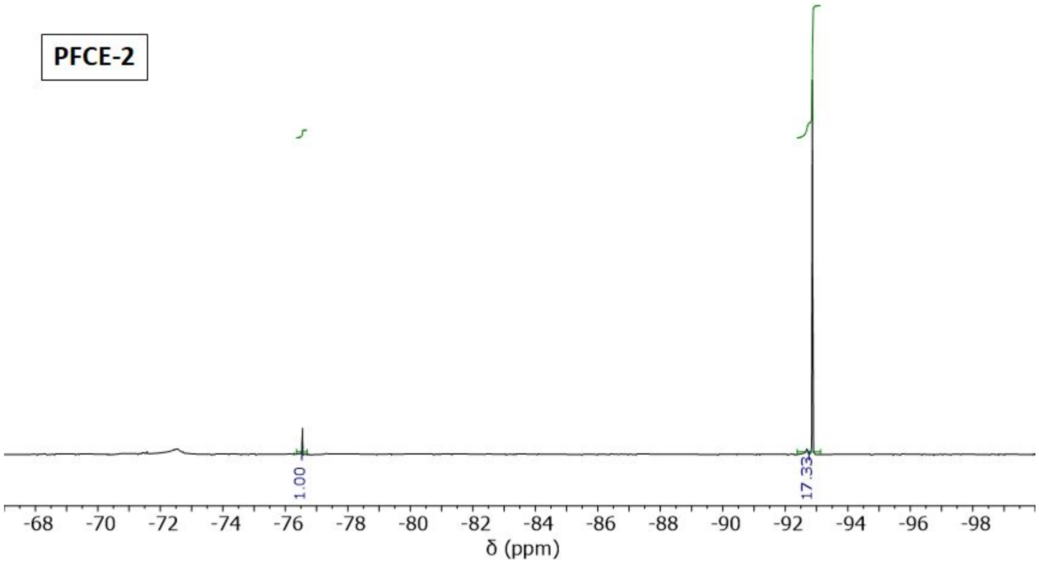

Figure S11. ^{19}*F-NMR spectra of* ***PFCE-2****.*

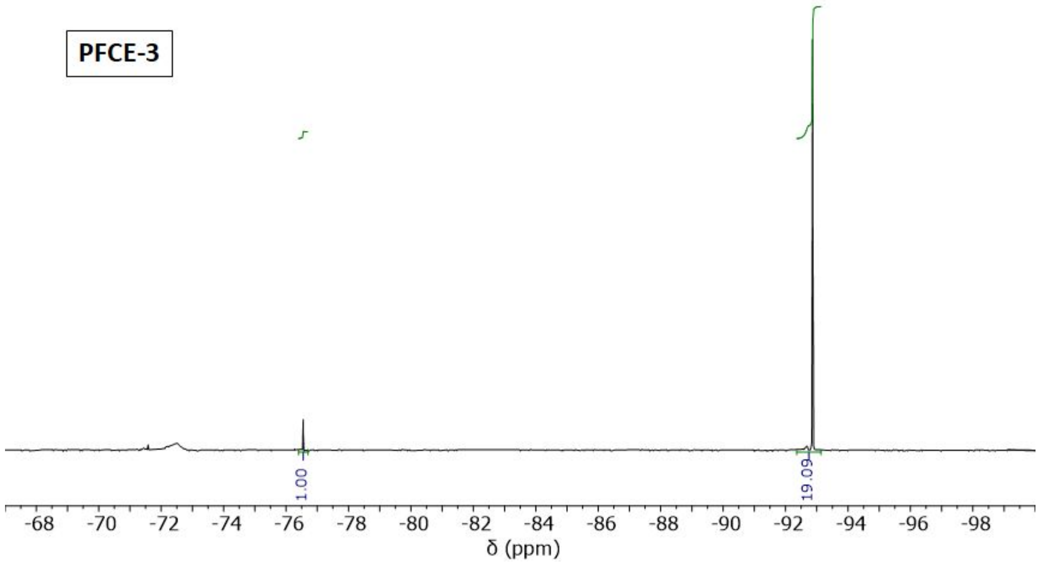

Figure S12. ^{19}*F-NMR spectra of* ***PFCE-3****.*

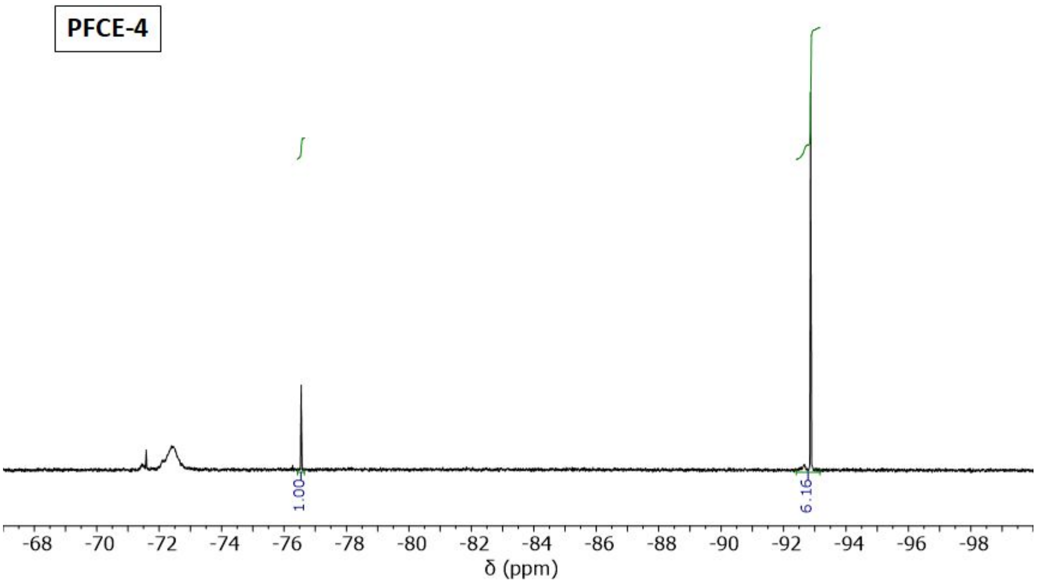

*Figure S13. ^{19}F-NMR spectra of **PFCE-4**.*

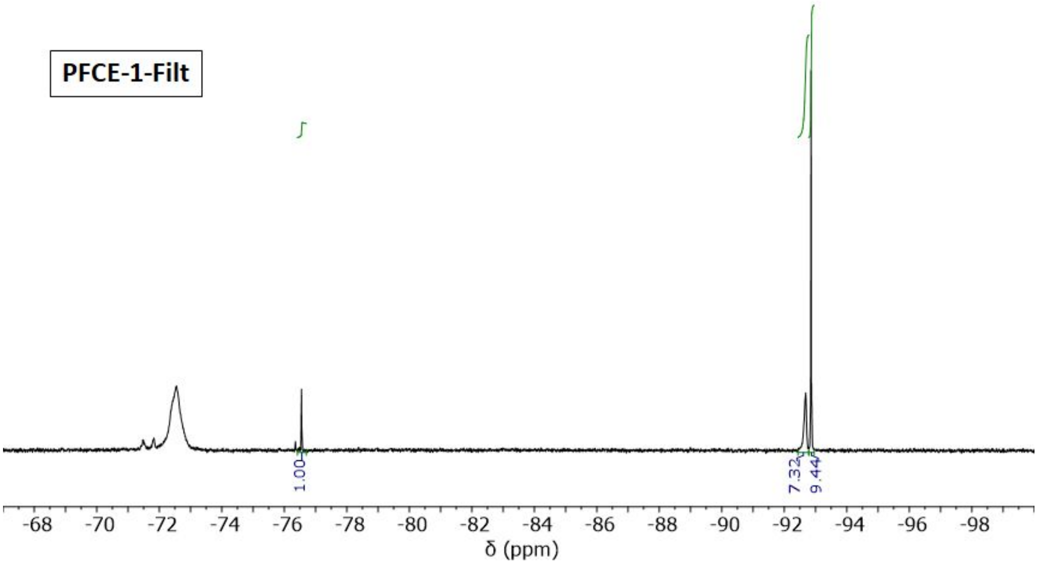

*Figure S14. ^{19}F-NMR spectra of **PFCE-1-Filt**.*

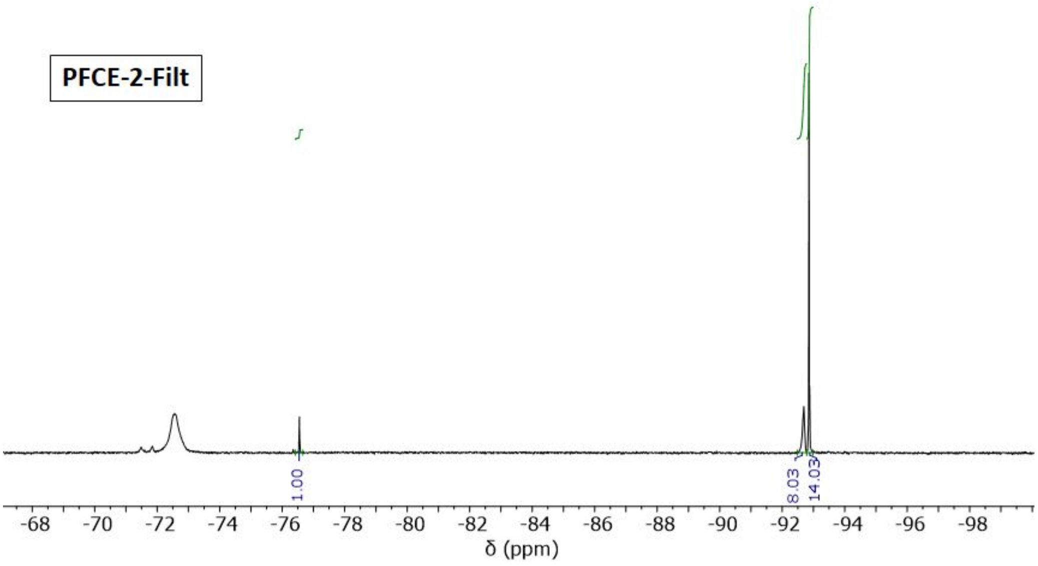

*Figure S15. ^{19}F-NMR spectra of **PFCE-2-Filt**.*

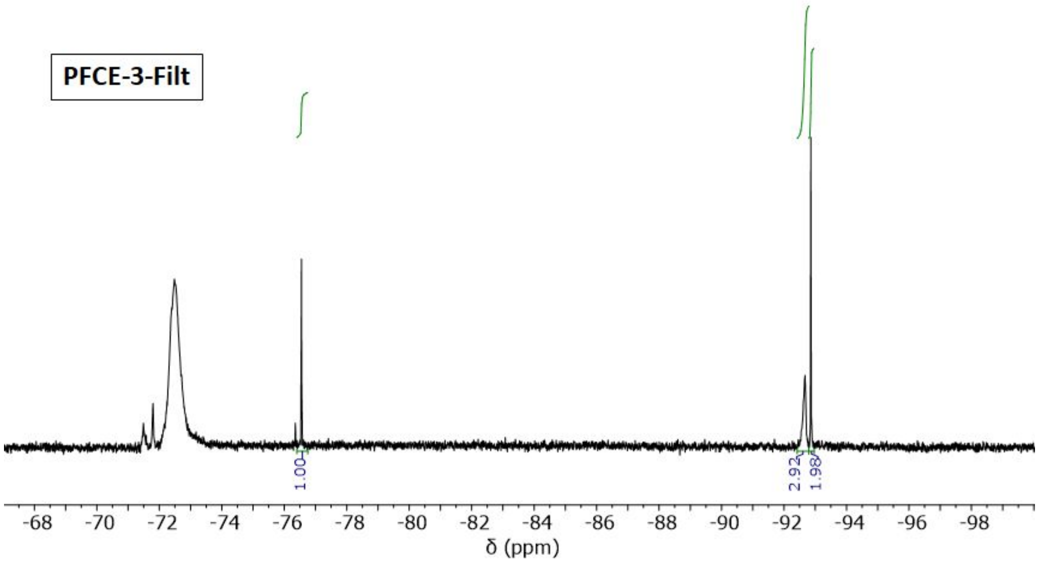

*Figure S16. ^{19}F-NMR spectra of **PFCE-3-Filt**.*

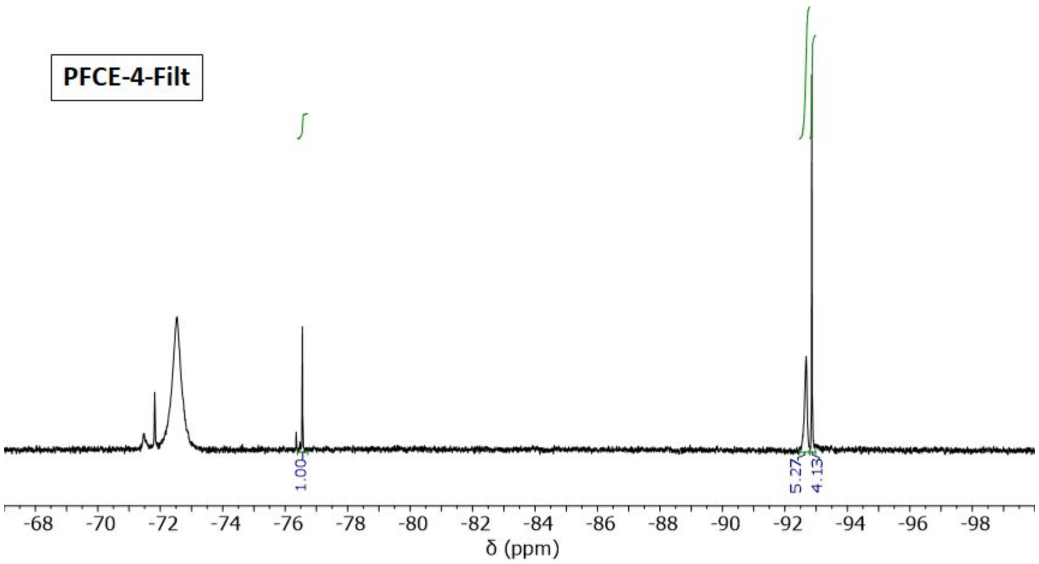

*Figure S17. ^{19}F-NMR spectra of **PFCE-41-Filt**.*

4.2. DLS analysis of PMA-100F coated PFCE

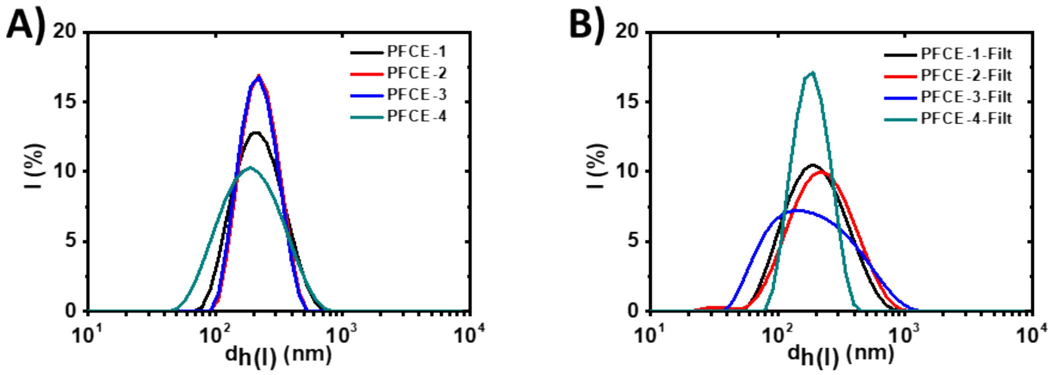

Figure S18. Size distributions in intensity of PFCE encapsulated by PMA-100F in water for A) fresh, non-filtered samples and B) filtered samples.

Table S8. Summary of d_h values and polydispersity as obtained by DLS.

Sample	d_h(I) (nm)	PdI	Sample	d_h(I) (nm)	PdI
**PFCE-1**	211.9 ± 11.1	0.23	**PFCE-1-Filt**	192.1 ± 4.2	0.18
**PFCE-2**	213.4 ± 11.3	0.18	**PFCE-2-Filt**	217.4 ± 14.6	0.26
**PFCE-3**	237.1 ± 14.4	0.21	**PFCE-3-Filt**	207.7 ± 7.3	0.27
**PFCE-4**	226.7 ± 3.7	0.16	**PFCE-4-Filt**	220.6 ± 13.0	0.40

4.3. Transverse and Longitudinal relaxation times (T2 and T1) measurement of fluorine

^{19}F T_2 measurements were performed using Bruker's standard CPMG sequence at 282.38 MHz with the following parameters: TR = 12.0s, 16 echo times covering a range between 0.02 and 3.67 s, SW = 20 ppm, NS = 20, DS = 4 and 32K points. ^{19}F T_1 measurements were performed using inversion recovery method with Bruker's t1ir sequence at 282.38 MHz MHz with the following parameters: TR = 8.0s, 16 inversion times covering a range between 5.5 and 0.001 s, SW = 20 ppm, NS = 20, DS = 4 and 32K points.

Table S9. Summary of measured T1 and T2 values for PFCE encapsulated in PMA-100F.

Sample	T_1 at -92.86 ppm (ms)	T_2 at -92.86 ppm (ms)	T_1 at -92.65ppm (ms)	T_2 at -92.65 ppm (ms)
**PFCE-1**	1038	888	-	-
**PFCE-2**	1034	832	-	-
**PFCE-3**	1061	862	-	-
**PFCE-4**	1062	834	-	-
**PFCE-1-Filt**	1064	840	847	186
**PFCE-2-Filt**	1010	890	676	195
**PFCE-3-Filt**	1039	841	765	96
**PFCE-4-Filt**	996	804	655	105

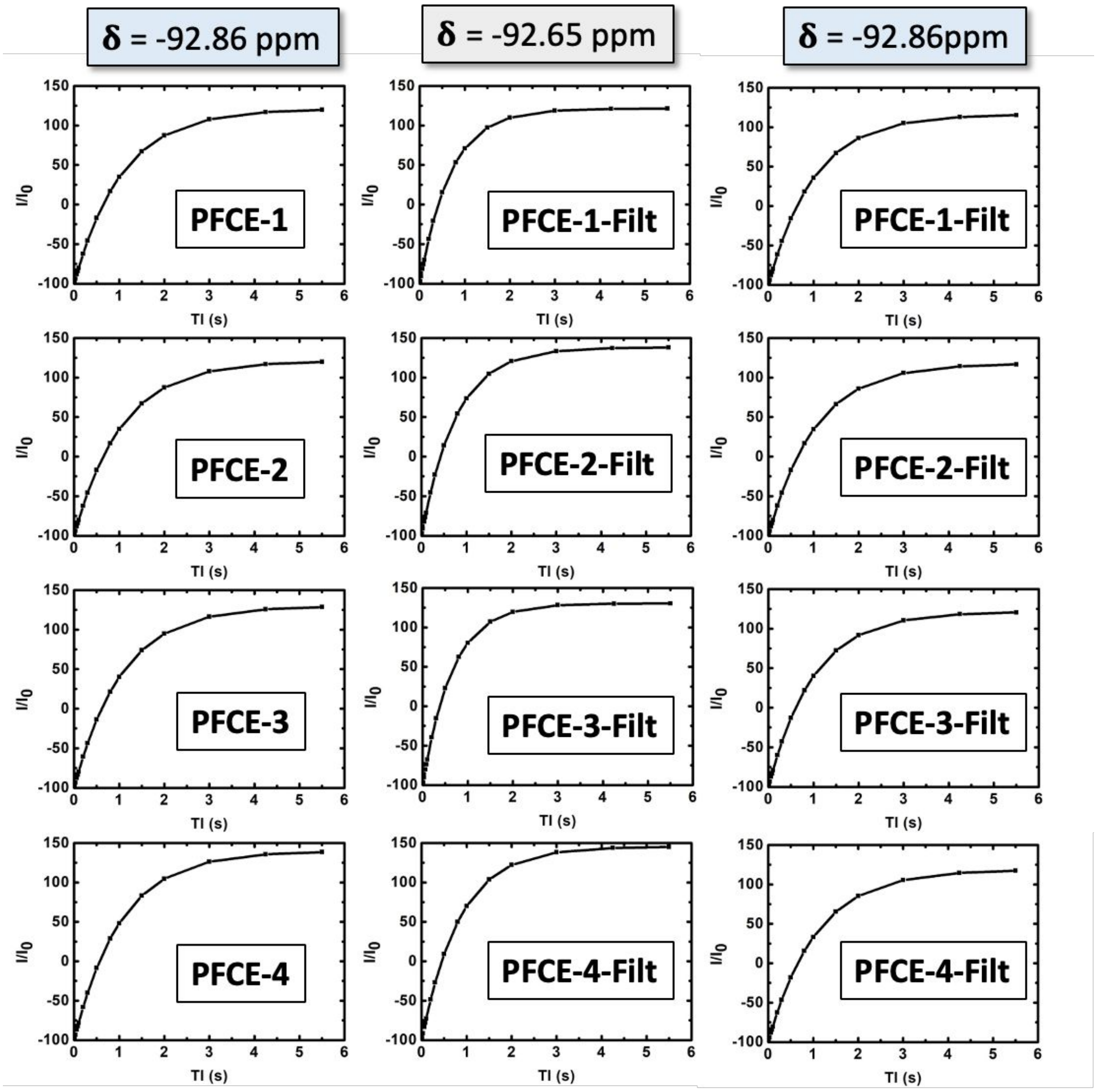

Figure S19. Normalized inversion recovery plots for PMA-100F coated PFCE before and after filtration through 0.45 µm syringe filter, for all the encapsulation conditions tested and for all the PFCE peaks analysed. TI is time of inversion.

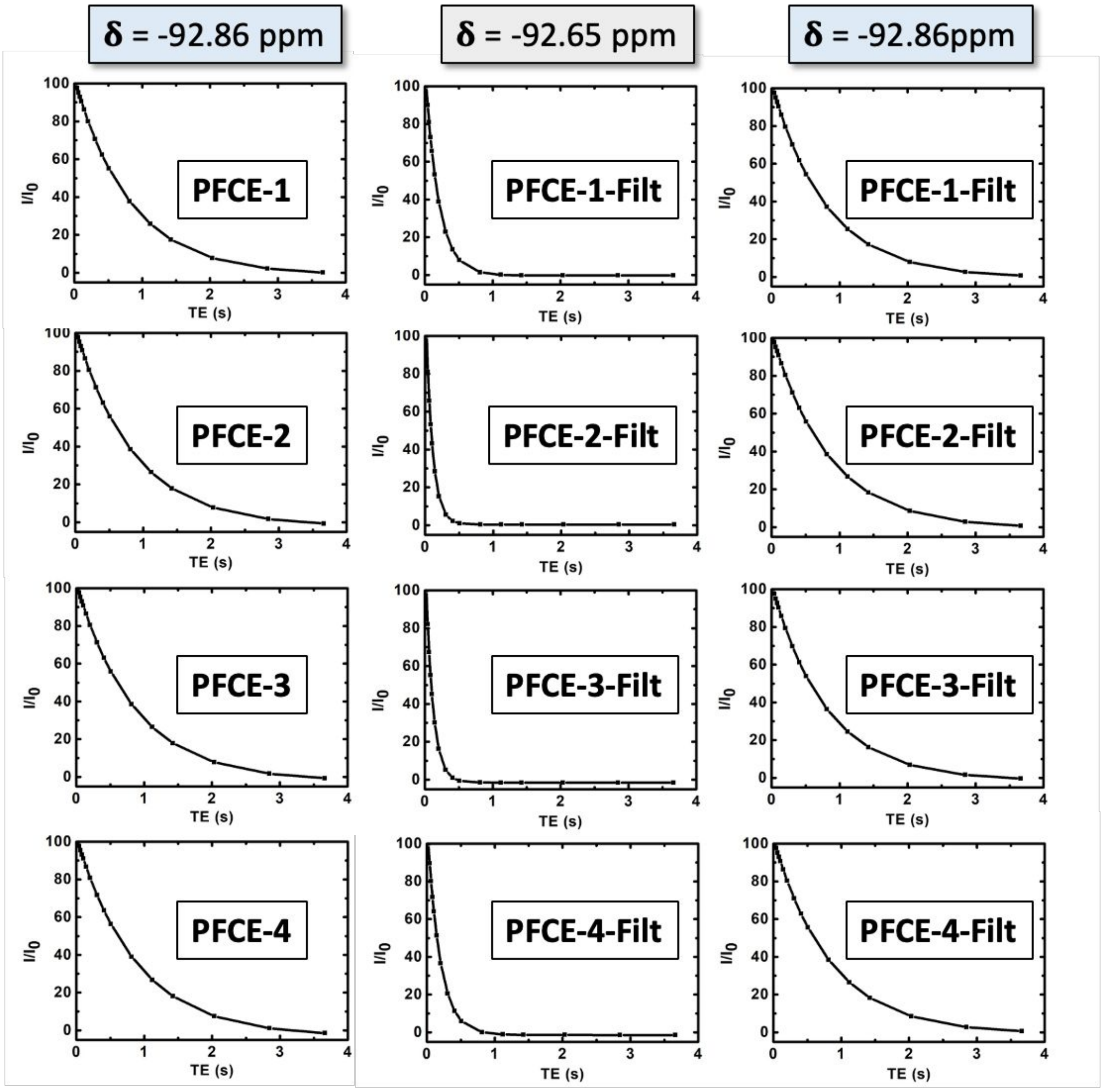

Figure S20. Normalized exponential decay plots for PMA-100F coated PFCE before and after filtration through 0.45 µm syringe filter, for all the encapsulation conditions tested and for all the PFCE peaks analysed. TE is time of echo.

5. Encapsulation and water transfer of PERFECTA

5.1. Synthesis of PERFECTA

PERFECTA.[2] Triphenylphosphine (3.172 g, 12.09 mmol) and pentaerythritol (279 mg, 2.05 mmol) were dissolved in anhydrous THF (8 mL) under nitrogen atmosphere. The mixture was cooled to 0 °C, and DIAD (2.4 mL, 12.10 mmol) was added dropwise. After the addition, the mixture was allowed to reach room temperature and stirred for 30 minutes. After this time, perfluoro *tert*-butanol (1.7 mL, 12.19 mmol) was added, and the resulting mixture was stirred at 45 °C for 72 hours. The mixture was then cooled to 0 °C, filtered and washed with THF, water and acetone to obtain PERFECTA as a white solid (409.2 mg, 20%).

Table S10. Amount of PERFECTA added and detected by ^{19}F NMR in different media.

Sample	PERFECTA added (µmol)	PERFECTA in H_2O (µmol)	Encapsulation efficiency (%)
**In NaOH 0.1 M**	10.32	8.28	80
**In H_2O**	10.12	5.63	56
**In $NaHCO_3$ 0.1 M, pH = 8.3**	10.00	4.01	40

[2] Tirotta, I.; Mastropietro, A.; Cordiglieri, C.; Gazzera, L.; Baggi, F.; Baselli, G.; Grazia Bruzzone, M.; Zucca, I.; Cavallo, G.; Terraneo, G.; Baldelli Bombelli, F.; Metrangolo, P.; Resnati, G. A Superfluorinated Molecular Probe for Highly Sensitive in Vivo 19F-MRI. *J. Am. Chem. Soc.* **2014**, *136*, 8524–8527.

5.2. ^{19}F-NMR of PERFECTA encapsulated in PMA-100F in NaOH 0.1M and after transfer to water or $NaHCO_3$ 0.1 M

A coaxial insert loaded with TFA in deuterium oxide was used as internal standard.

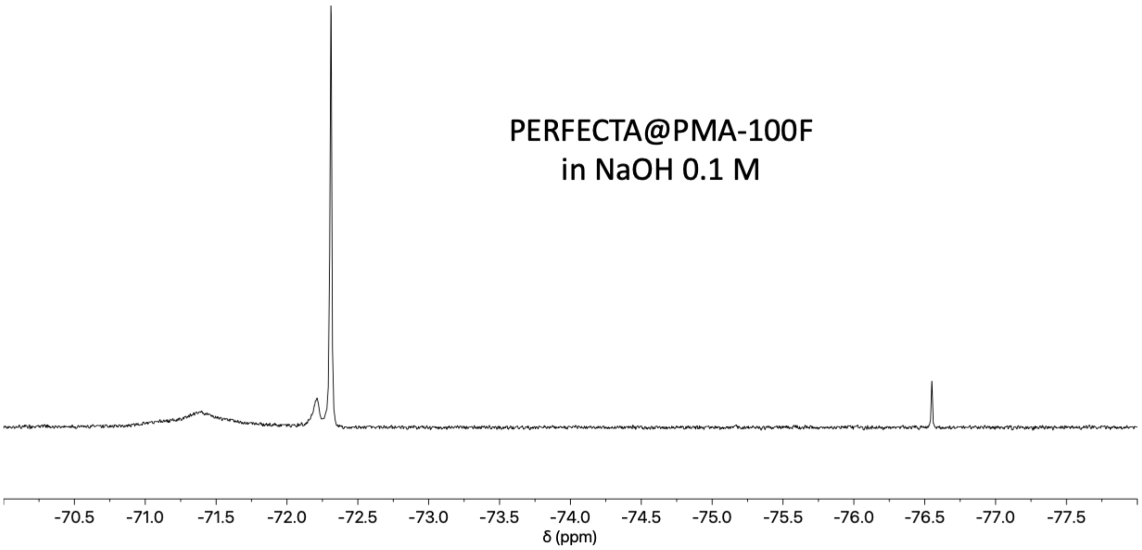

*Figure S21. ^{19}F-NMR spectra of **PERFECTA@PMA-100F** in NaOH 0.1 M.*

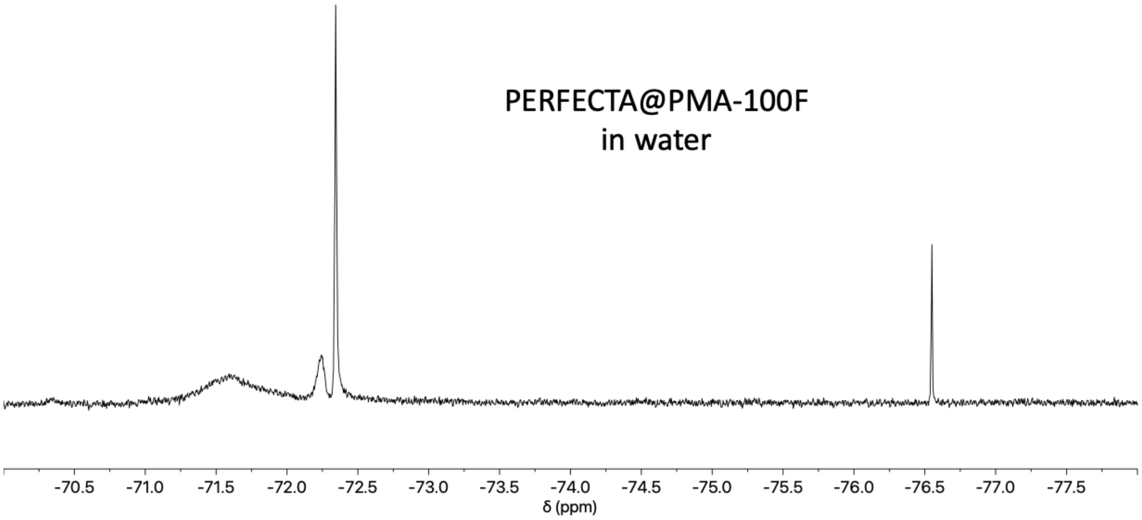

*Figure S22. ^{19}F-NMR spectra of **PERFECTA@PMA-100F** in water.*

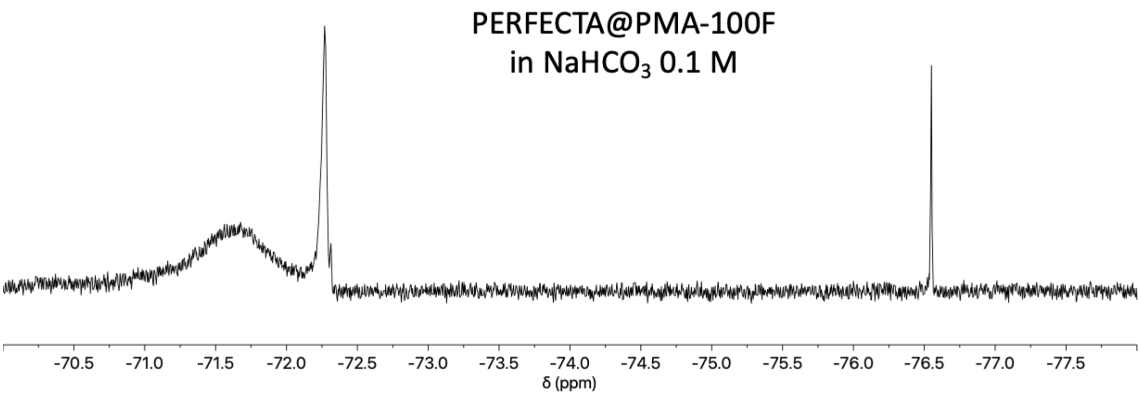

*Figure S23. ^{19}F-NMR spectra of **PERFECTA@PMA-100F** in $NaHCO_3$ 0.1 M.*

5.3. DLS analysis of PMA-100F coated PERFECTA

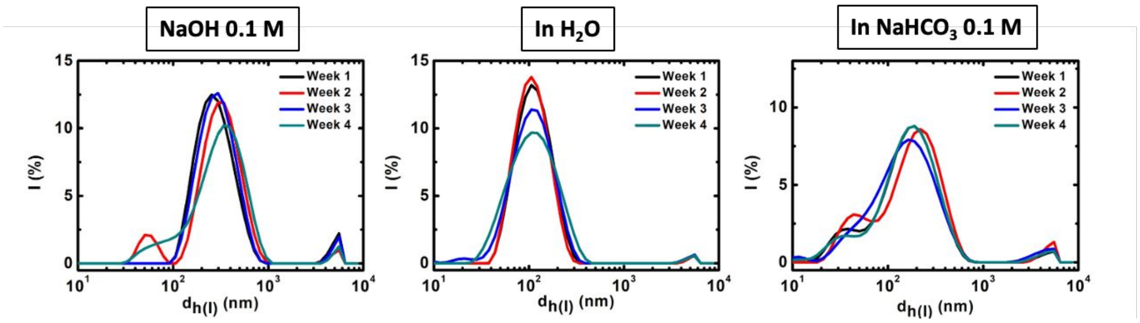

Figure S24. Size distributions in intensity of PERFECTA encapsulated with PMA-100F in NaOH 0.1 M, water and $NaHCO_3$ 0.1 M, respectively, in different weeks.

Table S11. Summary of d_h values and polydispersity as obtained by DLS.

Sample	d_h(I) (nm)	Polydispersity
**In NaOH 0.1 M, week 1**	321.8 ± 26.2	0.39
**In NaOH 0.1 M, week 2**	336.7 ± 19.8	0.42
**In NaOH 0.1 M, week 3**	306.0 ± 38.4	0.37
**In NaOH 0.1 M, week 4**	313.6 ± 16.4	0.36
**In H_2O, week 1**	116.1 ± 4.1	0.24
**In H_2O, week 2**	116.2 ± 3.2	0.26
**In H_2O, week 3**	122.6 ± 2.7	0.26
**In H_2O, week 4**	125.5 ± 2.0	0.27
**In $NaHCO_3$ 0.1 M, week 1**	197.5 ± 9.8	0.43
**In $NaHCO_3$ 0.1 M, week 2**	198.4 ± 17.1	0.41
**In $NaHCO_3$ 0.1 M, week 3**	189.9 ± 12.2	0.42
**In $NaHCO_3$ 0.1 M, week 4**	185.1 ± 11.2	0.44

5.4. Transverse and longitudinal relaxation times analysis of PERFECTA encapsulated in PMA-100F

Table S12. Summary of measured T_1 and T_2 values for PERFECTA encapsulated in PMA-100F.

Medium	T_1 at -72.3 ppm (ms)	T_2 at -72.3 ppm (ms)	T_1 at -72.2ppm (ms)	T_2 at -72.2 ppm (ms)
**NaOH 0.1 M**	532	247	508	145
**Water**	595	153	566	88
**$NaHCO_3$ 0.1 M**	519	247	-	-

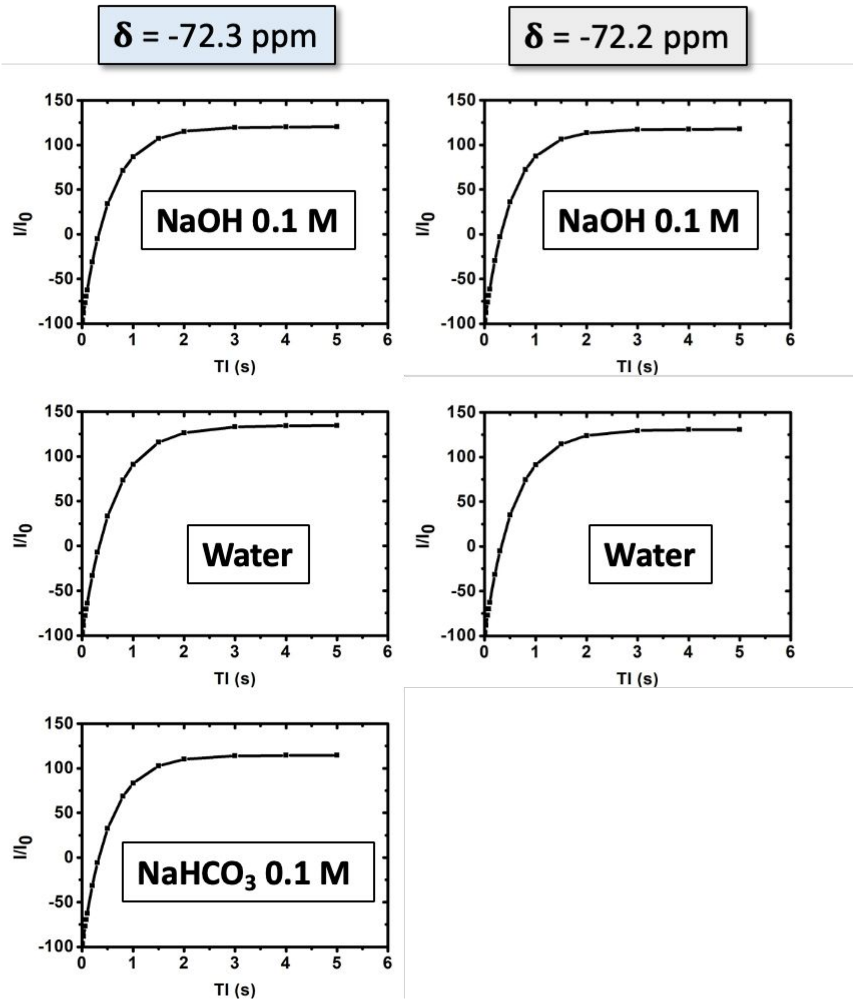

Figure S25. Normalized inversion recovery plots for PMA-100F coated PERFECTA for each media. TI = Time of Inversion.

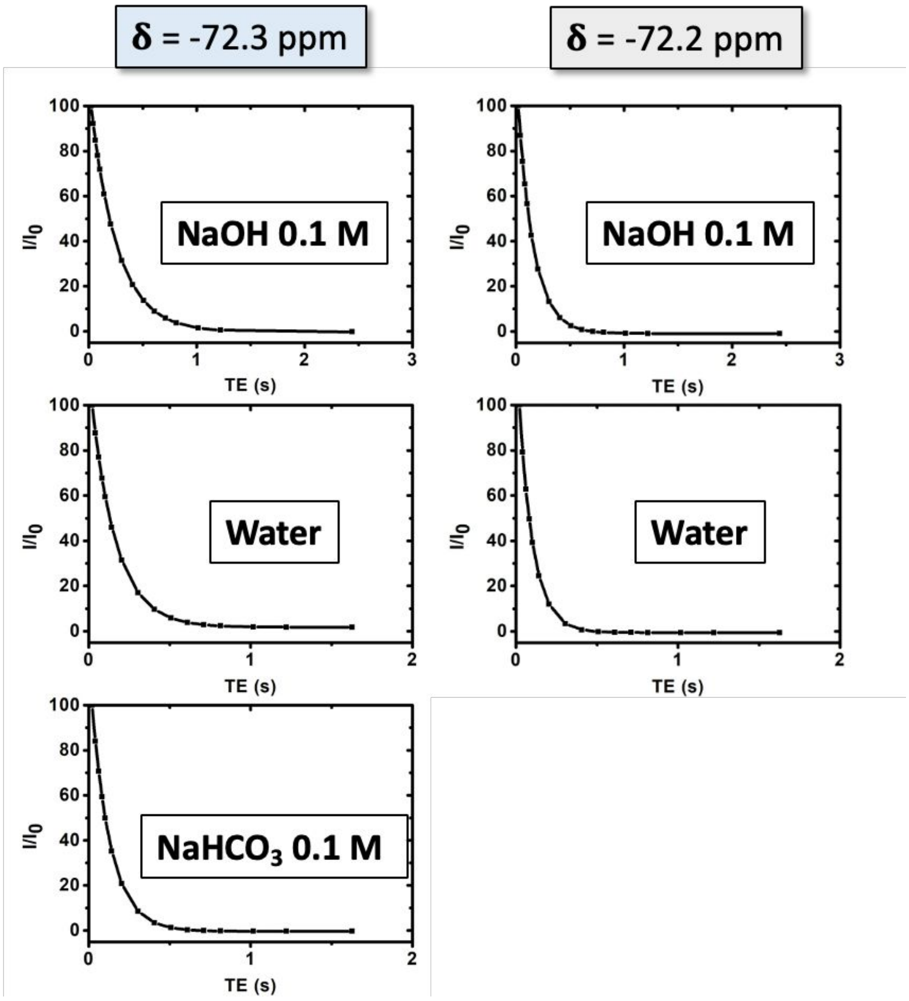

Figure S26. Normalized exponential decay plots for PMA-100F coated PERFECTA for each media. TE = Time of Echo.
